# Sensory coding and contrast invariance emerge from the control of plastic inhibition over emergent selectivity

**DOI:** 10.1371/journal.pcbi.1009566

**Published:** 2021-11-29

**Authors:** René Larisch, Lorenz Gönner, Michael Teichmann, Fred H. Hamker

**Affiliations:** 1 Department of Computer Science, Artificial Intelligence, TU Chemnitz, Chemnitz, Germany; 2 Faculty of Psychology, Lifespan Developmental Neuroscience, TU Dresden, Dresden, Germany; 3 Bernstein Center Computational Neuroscience, Berlin, Germany; Research Center Jülich, GERMANY

## Abstract

Visual stimuli are represented by a highly efficient code in the primary visual cortex, but the development of this code is still unclear. Two distinct factors control coding efficiency: Representational efficiency, which is determined by neuronal tuning diversity, and metabolic efficiency, which is influenced by neuronal gain. How these determinants of coding efficiency are shaped during development, supported by excitatory and inhibitory plasticity, is only partially understood. We investigate a fully plastic spiking network of the primary visual cortex, building on phenomenological plasticity rules. Our results suggest that inhibitory plasticity is key to the emergence of tuning diversity and accurate input encoding. We show that inhibitory feedback (random and specific) increases the metabolic efficiency by implementing a gain control mechanism. Interestingly, this led to the spontaneous emergence of contrast-invariant tuning curves. Our findings highlight that (1) interneuron plasticity is key to the development of tuning diversity and (2) that efficient sensory representations are an emergent property of the resulting network.

## Author summary

Synaptic plasticity is crucial for the development of efficient input representation in the different sensory cortices, such as the primary visual cortex. Efficient visual representation is determined by two factors: representational efficiency, i.e. how many different input features can be represented, and metabolic efficiency, i.e. how many spikes are required to represent a specific feature. Previous research has pointed out the importance of plasticity at excitatory synapses to achieve high representational efficiency and feedback inhibition as a gain control mechanism for controlling metabolic efficiency. However, it is only partially understood how the influence of inhibitory plasticity on excitatory plasticity can lead to an efficient representation. Using a spiking neural network, we show that plasticity at feed-forward and feedback inhibitory synapses is necessary for the emergence of well-distributed neuronal selectivity to improve representational efficiency. Further, the emergent balance between excitatory and inhibitory currents improves the metabolic efficiency, and leads to contrast-invariant tuning as an inherent network property. Extending previous work, our simulation results highlight the importance of plasticity at inhibitory synapses.

## Introduction

The primary visual cortex (V1) represents visual stimuli in a highly efficient manner [1, 2]. Recent research has identified two distinct factors underlying the efficiency of visual representations: First, representational efficiency in terms of absolute information content, which is mainly determined by the receptive field tuning diversity [3]. Second, metabolic efficiency in terms of the number of spikes required to represent a specific input stimulus. This aspect is strongly influenced by gain control mechanisms caused by inhibitory feedback processing [4, 5]. How these determinants of coding functionality are shaped is only partially understood. While it has long been known that excitatory plasticity is necessary for the development of an accurate and efficient input representation [6, 7, 8], there has recently been growing interest in the role of inhibitory plasticity, fueled by recent studies demonstrating plasticity at inhibitory synapses [9]. As the synaptic plasticity of inhibitory interneurons in V1 likely exerts strong effects on the outcome of excitatory plasticity [10], complex circuit-level interactions occur between both types of plasticity. This notion has received further support based on recent theoretical studies [11]. Above all, these findings raise the question of how excitatory and inhibitory plasticity can cooperate to enable the development of an efficient stimulus code.

Network models have proposed neural-level mechanisms of sparse code formation [6] based on Hebbian plasticity. However, these models typically rely on simplified learning dynamics [8, 12, 13] or consider plasticity only at a subset of projections in the network [14, 15], not addressing the development of feedback-based gain control. As such, it remains unclear how functional input encoding can emerge during development in a more detailed V1 circuit model.

We here propose how a single underlying mechanism—the influence of inhibitory plasticity on excitatory plasticity—is sufficient to explain both, the observed feed-forward tuning and neuronal gain-control by feedback processing, which we demonstrate in a spiking network model of V1-layer 4. To test for an additional influence of inhibitory strength on the emergence of feed-forward tuning, we varied the balance between excitation and inhibition in the network. Our findings support a role for inhibitory plasticity in the joint development of feed-forward tuning and balanced inhibitory feedback currents. Importantly, this balance leads to the spontaneous emergence of contrast-invariant tuning curves, as an inherent phenomenon of the network and its plasticity dynamics. Our results link both representational efficiency and metabolic efficiency to synaptic plasticity mechanisms.

## Results

**Fig 1:**
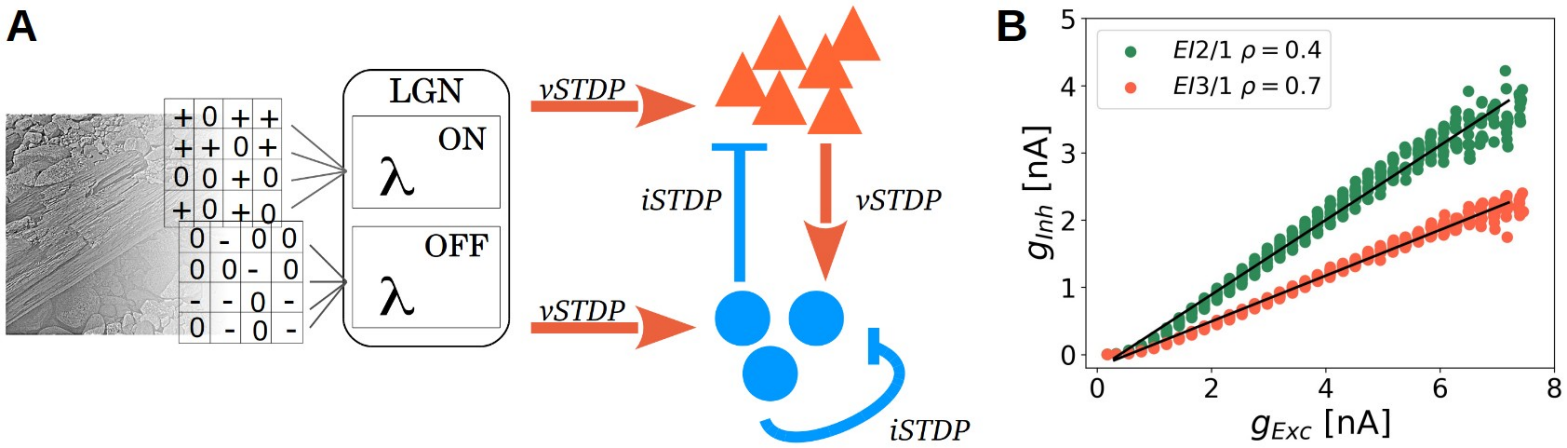
Network with excitatory and inhibitory plasticity rules. **A** Whitened image patches of size 12x12 were converted to Poisson spike trains by setting the firing rates of LGN ON- and OFF-populations to the positive and the negative part of the pixel values, respectively. Feed-forward inputs from LGN project both onto excitatory and inhibitory V1 populations, which are mutually connected. The circuit therefore implements both feed-forward and feedback inhibition. Inhibitory interneurons receive additional recurrent inhibitory connections. All excitatory synapses (orange) changes via the voltage-based STDP rule (vSTDP) [20]. All inhibitory synapses (blue) changes via the inhibitory STDP rule (iSTDP) [21]. Connectivity patterns are all-to-all. Population sizes are: LGN, 288 neurons; V1 excitatory, 144 neurons; V1 inhibitory, 36 neurons. Neurons in the LGN population show Poisson activity and are split into ON- and OFF- subpopulations. **B** Inhibitory currents as a function of excitatory currents, averaged across the duration of a stimulus. The post-synaptic target firing rate of the iSTDP rule (*ρ*) controls the excitation to inhibition ratio at excitatory cells. For the *EI*2/1 model (green dots) a value of *ρ* = 0.4 leads to higher inhibitory currents than *ρ* = 0.7 for the *EI*3/1 model.

To investigate the interaction between excitatory and inhibitory plasticity, we designed a spiking network model of V1-layer 4 consisting of an excitatory and inhibitory population, stimulated with natural image patches (Fig 1A) (see **Network input**). The circuit of our neuronal network implements both feed-forward and feedback inhibition, in agreement with anatomical findings [5]. Although different kinds of inhibitory neurons have been found in the neocortex [16, 17], our network contains only one population of inhibitory neurons, as a simplification. The size of the inhibitory population was chosen to match the 4:1 ratio between excitatory and inhibitory neurons found in striate cortex [16, 18, 19]. The plasticity of the excitatory synapses follows the voltage-based triplet spike timing-dependent plasticity (STDP) rule proposed by Clopath et al. [20]. The strength of the inhibitory synapses changes according to the symmetric inhibitory STDP rule described by Vogels et al. [21], which achieves homeostasis by maintaining a constant postsynaptic firing rate (*ρ*).

To analyze the influence of inhibitory plasticity on excitatory plasticity, we used two approaches. First, we investigate how the balance between excitation and inhibition influences the emergence of neuronal gain-control and feed-forward tuning, by comparing a network with a 2 : 1 ratio of excitation to inhibition (E/I ratio) to a model version with a 3 : 1 E/I ratio, averaged on 10, 000 natural scene patches (Fig 1B). This ratio is adjusted via the *ρ* parameter exclusively. Additionally, we blocked inhibitory synapses after learning to investigate the dynamic effects of inhibition on network coding (called *blockInh*). To analyze the influence of inhibition during learning after all, a further model configuration did not contain any inhibitory synapses (called *noInh* model) and learns with the absence of inhibition.

Second, to analyze if plastic inhibition has a measurable effect during learning, we deactivated plasticity selectively at specific connections for two model variations: Only at the inhibitory feedback connections (called *fix fb inh*) and at all excitatory projections to the inhibitory population (called *fix ff inh*). We used shuffled weight matrices from a successfully learned *EI*2/1 model for all connections to ensure that the network will have an E/I ratio comparable to networks where all synapses are plastic. Only the incoming excitatory weights of the excitatory population are chosen anew from a normal distribution. To verify that learning is successful with the shuffled pre-learned weights, we trained one model variation where all connections are plastic (see S1 Fig). While we vary the inhibitory influence, the feed-forward synapses to the excitatory population are plastic in all model configurations.

In all model configurations, the populations consist of the same number of neurons and synapses between them. Each model configuration was repeated 20 times. If not mentioned otherwise, initialized with randomly chosen weight values, to test the stability and reproducibility of the observed outcomes.

We first analyze the structural characteristics of the network as a consequence of the learning process, and then present its functional properties. In both cases, we investigate the effect of plastic vs fixed synapses and different inhibitory strengths.

### Emergence of diversely tuned receptive fields

**Fig 2:**
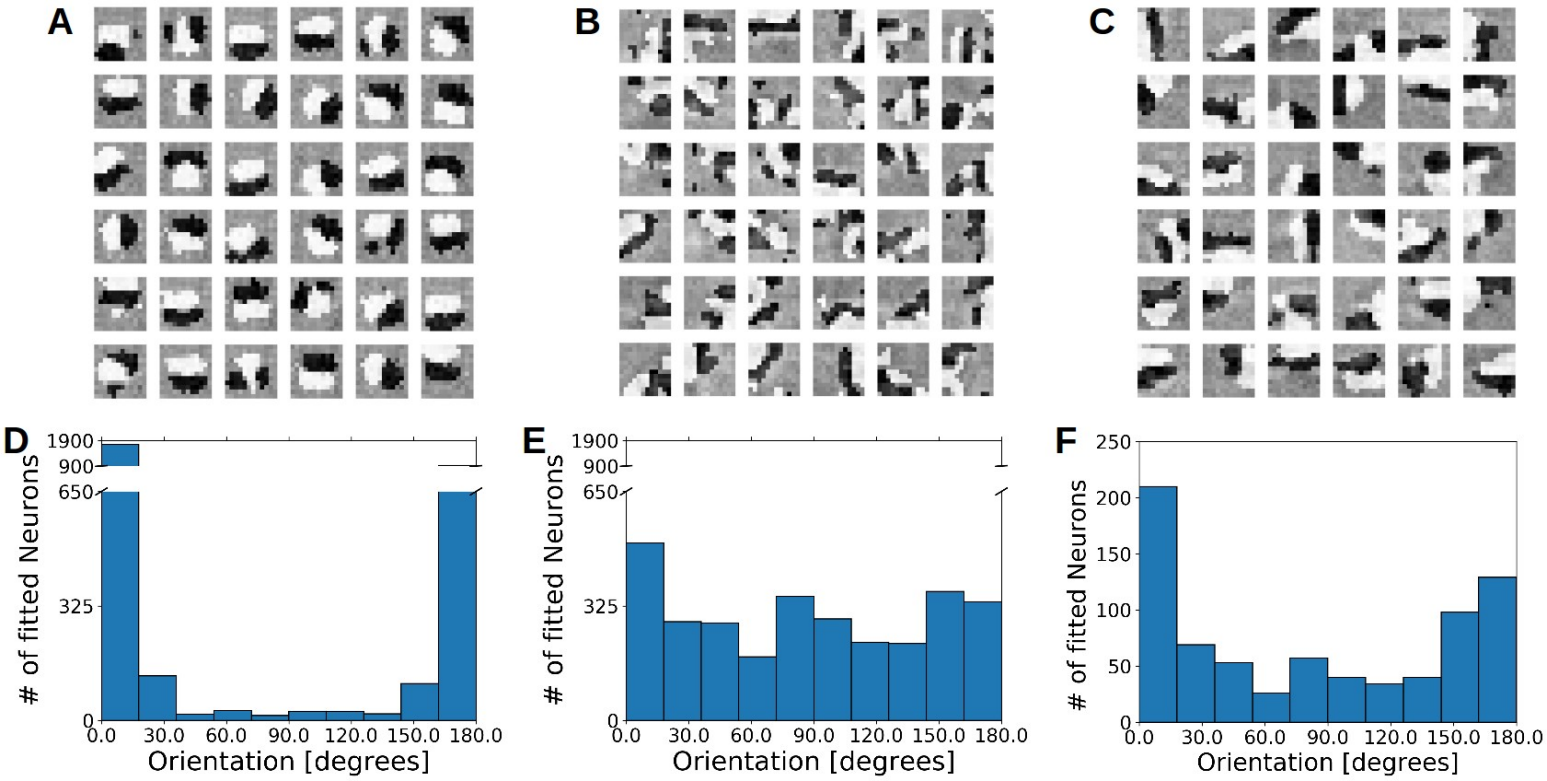
Tuning diversity requires inhibition during learning. **A–C** Learned response profile of 36 excitatory neurons from the *noInh* model **A**, of 36 excitatory neurons from the *EI*2/1 model **B**, and of all 36 inhibitory neurons from the *EI*2/1 model **C**, measured with the spike triggered average method. Lighter pixels represent positive values and darker values represent negative values. **D–F** Histogram of the spatial orientation across 20 model runs, of the *noInh* model’s excitatory population **D**, the *EI*2/1 model’s excitatory population **E**, and the the *EI*2/1 model’s inhibitory population **F**. Note the strong clustering of orientations in the noInh model **D**. The spatial orientation are measured by presenting sinus grating on different orientations (see **Tuning curves and orientation selectivity**).

The receptive fields of V1 simple cells are often described by Gabor functions [22, 23, 24]. We observe the emergence of Gabor-like receptive fields in our network for the excitatory and inhibitory population with the spike triggered average method (STA, see **Receptive field mapping**). Without inhibition, most of the receptive fields have a similar orientation and position (Fig 2A), as it is to be expected from the chosen learning rule, see also [20]. In contrast, the presence of plastic inhibition during learning resulted in a higher diversity of receptive fields with a more complex structure for the excitatory population (Fig 2B) and the inhibitory population (Fig 2C).

We observed the emergence of stable receptive fields after presenting approx. 200, 000 stimuli (see S2 and S3 Figs). We presented another 200, 000 stimuli to ensure that all synapses reach a stable state. The measured receptive fields showed a strong tendency for weight values to cluster around the minimum or the maximum value (see S4 Fig). This is a known characteristic of the learning rule chosen for excitatory synapses, which enforces strong synaptic competition [15, 20].

To measure the preferred orientation of each neuron, we presented sinus gratings with different orientations (see **Tuning curves and orientation selectivity**). To quantify the diversity of receptive field orientations across model repetitions, we calculated an orientation diversity index (*ODI*) via the Kullback-Leibler divergence between the measured orientations and an idealized uniform distribution of orientations (see Eq 15). Our calculated *ODI* is the exponential function of the Kullback-Leibler divergence and thus, higher values indicate a more uniform orientation distribution, which means a higher orientation diversity (see **Orientation diversity**).

**Fig 3:**
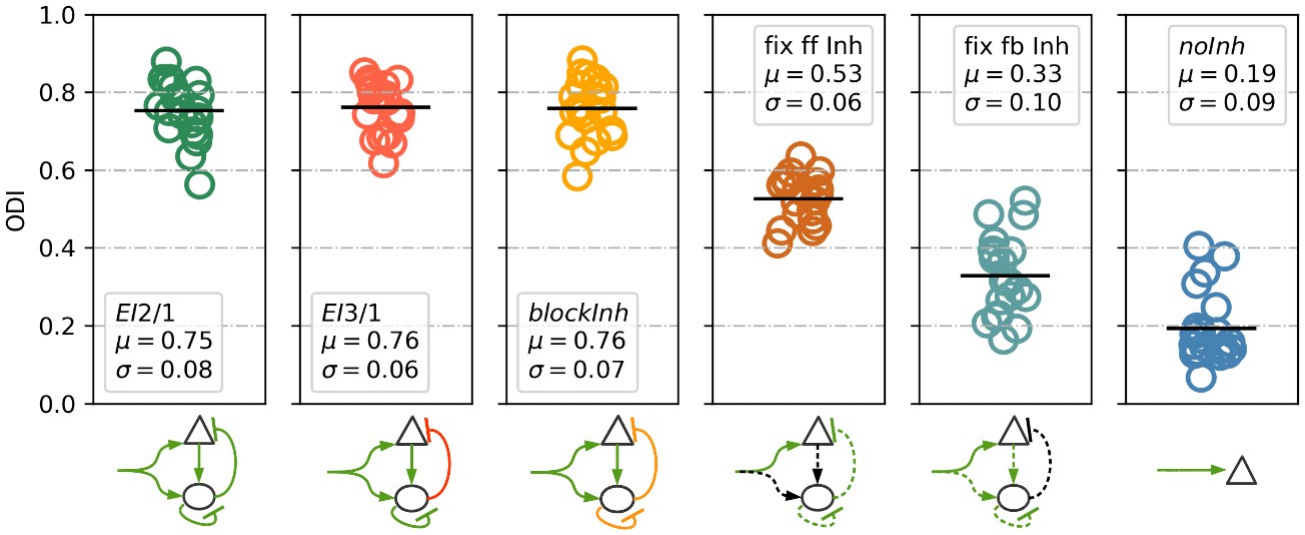
Tuning diversity is improved by plastic feed-forward and feedback inhibition. The orientation diversity index (*ODI*) is calculated via the Kullback-Leibler divergence between an idealized orientation distribution and the measured distribution in the network. The exponential of the divergence value is taken, so that higher values indicate a more uniform orientation distribution. Green arrows indicate plastic synapses, black arrows denote fixed synapses. Orange arrows indicate weaker synaptic connections. Dotted lines indicate that the model weights were initialized with shuffled values from weights of a previous run of the *EI*2/1 model. The highest diversity of RFs is observed for all models with fully plastic inhibition during learning (*EI*2/1, *EI*3/1, and *blockInh* models). Abolishing plasticity at feed-forward inputs to inhibitory neurons led to a moderate decrease of orientation diversity (*fixffinh* model). Blocking plasticity at inhibitory feedback synapses onto excitatory neurons led to a stronger decrease in orientation diversity (*fixfbinh* model). The lowest diversity was observed in the *noInh* model, were inhibition was fully absent.

A broader range of orientations emerged in the networks with inhibition (Fig 2E). Without inhibition, most receptive fields converge to a preferred orientation around 0° or 180° (Fig 2D). In the model with weaker inhibition (*EI*3/1), receptive fields converge to a very similar orientation distribution than in the *EI*2/1 model (see S5 Fig). This is mirrored in the orientation distribution (Fig 3). These results suggest that the presence of inhibition is more important for the emergence of receptive field diversity than its strength. In earlier studies of simple cells in the cat visual cortex, a broad distribution of different oriented simple cells has been reported, with a tendency to more cells selective for horizontal stimuli [25], vertical stimuli [26] or both [27]. In our simulations, both models with inhibition (*EI*2/1 and *EI*3/1) show a broad distribution with a slightly higher number of cells with a preference for horizontal and vertical stimuli (see Fig 2E and S5 Fig).

In addition, the inhibitory cells in the *EI*2/1 models also become selectively tuned, with a clear preference at 0° and 180° (Fig 2F), as well as the inhibitory cells in the *EI*3/1 models (see S5 Fig). This is in line with recent experimental reports of tuned inhibition in ferret V1 [28]. However, it is still debated whether tuned inhibition is as a general property of the visual system. For example, in mouse V1, recent research has identified inhibitory interneurons which are non-selective for orientation [29, 30, 31], very broadly tuned interneurons [32], and some subtypes of inhibitory interneurons which have a sharp tuning [33].

To further analyze the influence of fixed and plastic feed-forward and feedback inhibition on the resulting orientation diversity, we used the shuffled weight matrices from a *EI*2/1 model to ensure a comparable balance between excitation and inhibition, except for the feed-forward synapses of the excitatory cells, which are newly chosen from a normal distribution. We observed a reduction of tuning diversity in the *fix ff inh* model, in which the excitatory input weights to the inhibitory cells are unspecific and kept fixed (Fig 3). This presumably led to highly homogeneous activity across the interneuron population. A stronger reduction of tuning diversity occurred in the *fix fb inh* model, in which the inhibitory feedback connections were kept fixed. As a consequence, all excitatory neurons received unspecific inhibitory feedback. As expected, the *noInh* model showed the lowest degree of tuning diversity in the absence of any inhibition.

### Emergence of structured feed-forward and recurrent connectivity

**Fig 4:**
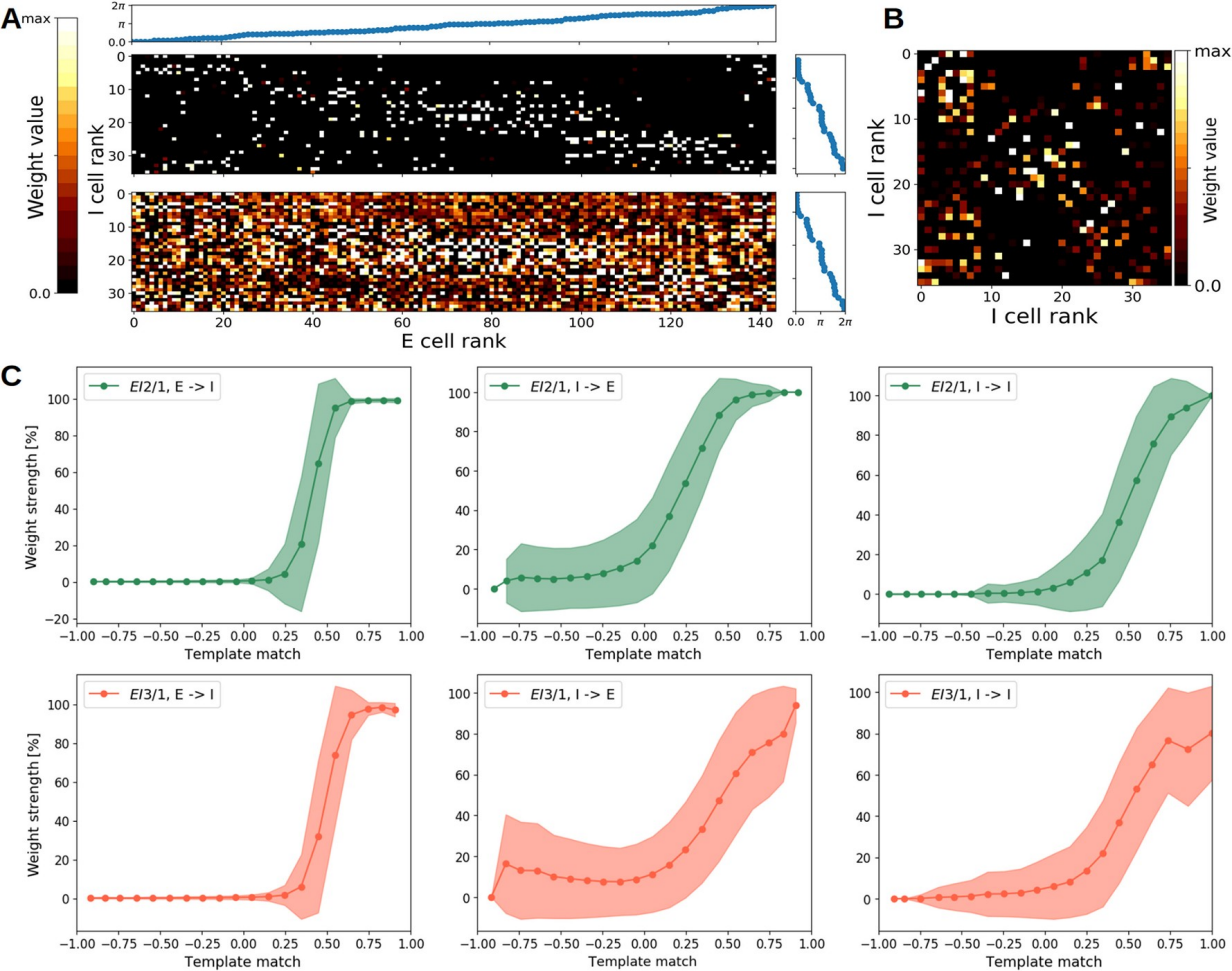
Synaptic connections reflect tuning similarity. Weight matrices from the excitatory to the inhibitory population (and vice versa) **A**, sorted by the receptive field orientation, and for the lateral inhibitory weights **B**. **A**, Top: Weights from the excitatory to the inhibitory population. **A**, Bottom: Weights from the inhibitory to the excitatory population. For display, all weight matrices were normalized by the maximum value. All weights are from the *EI*2/1 model. **C** Normalized synaptic strength as a function of the template match between the pre- and postsynaptic neuron’s receptive fields for the *EI*2/1 (first row) and the *EI*3/1 (second row) model. Shaded areas denote the mean +/- standard deviation. As expected, we observed strong weights between neurons with highly similar receptive fields, and near-zero weights between neurons with highly dissimilar receptive fields. For neurons with a moderate degree of RF similarity, we observed a steep transition from weak to strong weights at the E-I projection. At the I-E and I-I projections, this transition was more gradual.

As both, the excitatory and inhibitory cells in our network developed a tuning for orientation and position, we expected that their modifiable synaptic connections developed a specific pattern reflecting activity correlations [13, 14]. For an exemplary model simulation, our analysis confirmed that excitatory neurons developed strong connections to inhibitory neurons with similar orientation tuning (Fig 4A, top). Inhibitory weights to the excitatory layer showed a similar pattern, although with somewhat reduced specificity (Fig 4A, bottom). This implements an indirect inter-neuron connection between two excitatory neurons via mutually connected inhibitory neurons, to inhibit each other maximally. The development of recurrent inhibitory synapses between similarly tuned inhibitory cells can be observed as well (Fig 4B).

We next analyzed the connectivity structure based on all model repetitions as follows: First, for any pair of neurons sharing a synaptic connection, we calculated the template match between their receptive fields. Second, we binned the weight values and template match values for all neuron pairs from all model repetitions. Finally, we plotted the average weight strength as a function of the average template match for all neuron pairs per bin (Fig 4C). For both models with plastic inhibition (the *EI*2/1 and the *EI*3/1 model), we observe that neurons with a more similar receptive field have a higher mutual synaptic weight value. These results are in agreement with recent experimental reports from mouse visual cortex [34].

### Inhibition controls response decorrelation

**Fig 5:**
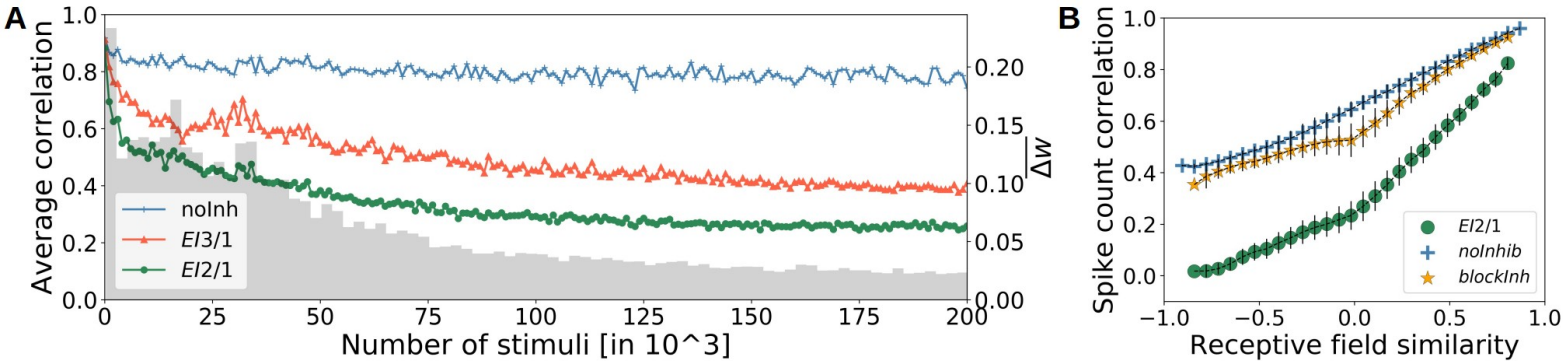
Inhibitory strength influences the response decorrelation. **A** The development of mean response correlation and weight change at the LGN excitatory synapses across learning. Stronger inhibition, in the *EI*2/1 model, leads to a stronger decorrelation of the neuron responses during learning (compare green with red (*EI*3/1) line). Mean response correlation changed only very slightly without inhibition (blue line). The change in the synaptic weights (gray bars) decreases over the developmental process, indicating the emergence of stable receptive fields. **B** Response correlation is higher for neurons with more similar receptive fields. Blocking inhibition (yellow line) after learning reveals that inhibition leads to a overall decrease of the response correlation (green line).

We observed that the different levels of inhibition in the *EI*2/1 and *EI*3/1 models led to similar orientation distributions. To investigate if response correlations between neurons only depend on the orientation similarity or whether lateral inhibition has an additional decorrelation effect (as mentioned in previous modeling approaches [8, 12, 13, 35]), we analyzed the development of correlations during receptive field learning (Fig 5A). During the first 250, 000 of all 400, 000 input stimuli, a weak reduction of the correlation can be observed in the *noInh* model. The *EI*2/1 model showed a pronounced decrease of correlations across learning, with the highest reduction occurring in the early phase of learning showing the highest amount of changes of the feed-forward weights. Weaker feedback inhibition (*EI*3/1 model) led to weaker decorrelation of neuronal activity. The researcher in [36] recorded the neuronal activity in V1 of macaque monkeys during the presentation of drifting sinusoidal gratings and reported a dependence of pairwise response correlations on orientation tuning similarity. We performed a similar analysis of our model data, to analyze the effect of feedback inhibition on the response correlation with respect to the orientation selectivity. We sorted all cell pairs by similarity, grouped them into 30 equally-spaced bins, and averaged their response correlation values within each bin, based on natural scene stimuli (Fig 5B). In both models without inhibition, we observed a mean response correlation of ≈ 0.95 for cell pairs with highly similar receptive fields. With inhibition, this value dropped to ≈ 0.8. By contrast, cell pairs with dissimilar receptive fields showed average correlation values of around 0.4 for the *noInh* and the *blockInh* model. Here, inhibitory processing substantially reduced the mean correlation to near zero-values for the *EI*2/1 model. A comparison between the *EI*2/1 model and its counterpart with blocked inhibition shows that dissimilarly tuned neuron pairs are more strongly decorrelated than pairs with highly similar tuning. At a first glance, this pattern contrasts with the emergent connectivity structure: The connectivity pattern favors strong mutual inhibitory connections between inhibitory neurons which receive projections from (and project back to) excitatory neurons with similar tuning, creating strong reciprocal inhibition (Fig 4A and 4B). However, our observation of target-specific decorrelation is best understood by considering that correlated spike counts can arise both through a similarity of tuning and through unspecific baseline activity, caused by contrast differences. Natural image patches are likely to evoke broad excitation among many cells, leading to different neuronal responses as sinusoidal gratings [37]. Due this, studies measuring the pairwise response correlation with sinusoidal gratings, reported a stronger decorrelation effect between similar neurons [36, 38]. Despite that, studies presenting natural scene inputs to measure the neuronal response correlation reported higher correlation values in comparison to sinusoidal gratings [1, 39] or more similar values [40]. The correlation between dissimilarly tuned neurons is most likely caused by the activity baseline, which is strongly reduced by inhibition. Besides, similarly tuned cells will retain strongly overlapping tuning curves even after reduction of unspecific activity, associated with strong correlation of their mean response [41]. Our observation that blocking the inhibitory processing leads to an overall increase of activity correlation is in line with previous studies. Sippy and Yuste [42] reported an increase of activity correlation between principal cells from 0.31 up to 0.66 by reducing inhibition pharmacologically in thalamocortical slices from mice (without considering receptive field similarities). A similar increase is observable if the compare the mean pairwise correlation from the *EI*2/1 model (0.32) and the *blockInh* counterpart (0.60).

### Inhibitory feedback shapes tuning curves

**Fig 6:**
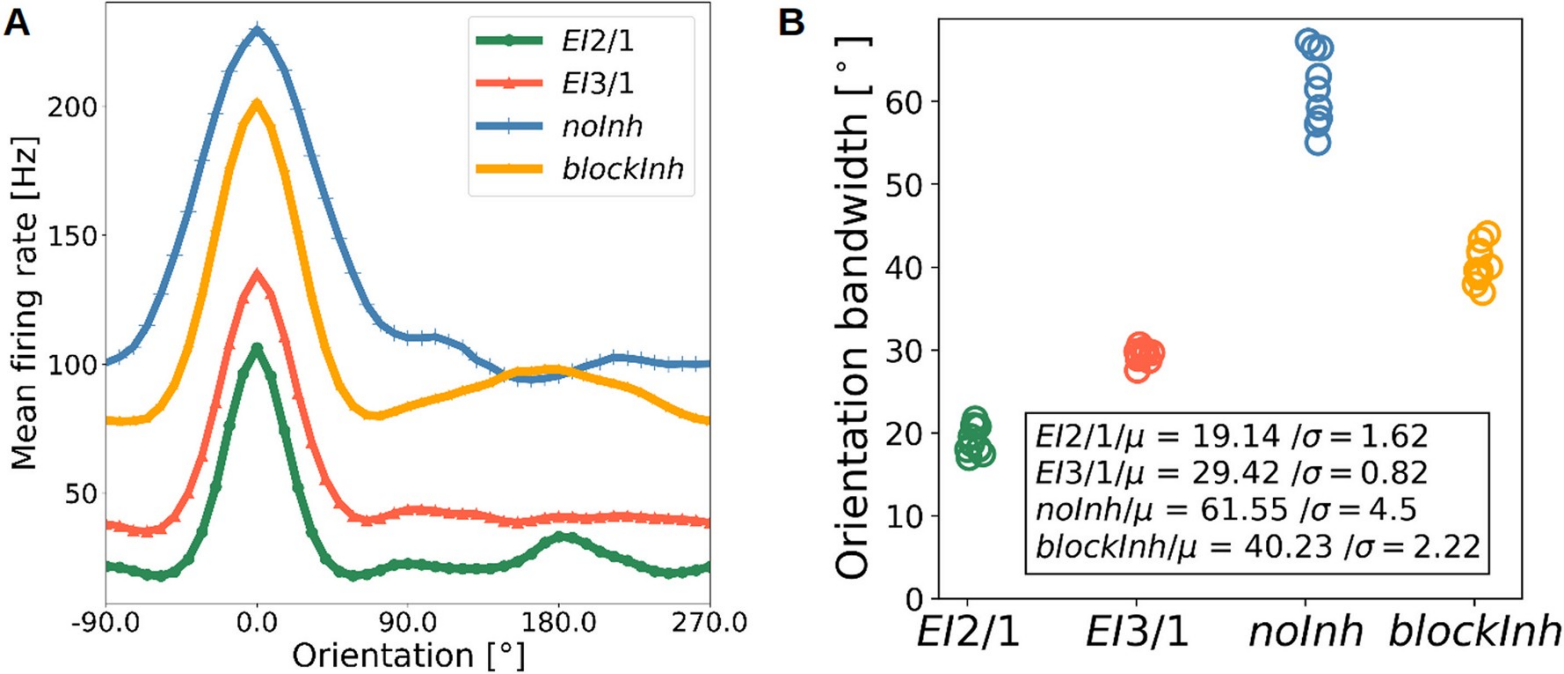
Inhibition controls tuning curve sharpening. **A** Average tuning curve of all excitatory cells in the *EI*2/1 model, the corresponding counterpart with blocked inhibition, and the no inhibition model. **B** The orientation bandwidth (OBW) of cells in all three models. Every point represents the average OBW resulting from model simulation. Smaller OBW values correspond to narrower tuning curves. As expected, the *EI*2/1 model (green) shows the narrowest tuning curves. The slightly reduced inhibitory strength in the *EI*3/1 model (red) leads to moderately broader tuning curves. Fully blocking inhibition post-learning leads to both wider tuning curves and increased baseline activity in the *blockInh* model (yellow). The broadest tuning curves and highest baseline activity were observed in the *noInh* model (blue), which produced relatively large receptive fields.

To quantify the effect of inhibition on the magnitude of individual neuronal responses, we measured orientation tuning curves of each neuron by sinusoidal gratings. For all approaches and model variants, the maximum firing rate in the input was set to ≈ 85*Hz* to obtain sufficiently high activity levels. We observed high baseline and peak activity in both model variants without inhibition (Fig 6A). However, activity levels in the *blockInh* model were lower than in the *noInh* model, likely owing to its smaller and more dispersed receptive fields. As expected, the model with active inhibitory feedback showed the lowest firing rate to input ratio. To obtain a measure of tuning sharpness, we next estimated the orientation bandwidth (OBW) of the excitatory population, based on the measured tuning curves. As expected, and consistent with previous observations [5, 43], our model shows a sharpening effect through inhibition (Fig 6B).

Duo to the same overall magnitude of inhibitory feedback as for the *EI*2/1 model, we assume for the *fix ff inh* and the *fix fb inh* a highly similar behavior, as it has been reported in previous work that broad or untuned inhibition causes tuning sharpening [17, 44, 45].

### Spontaneous emergence of contrast-invariant tuning curves

**Fig 7:**
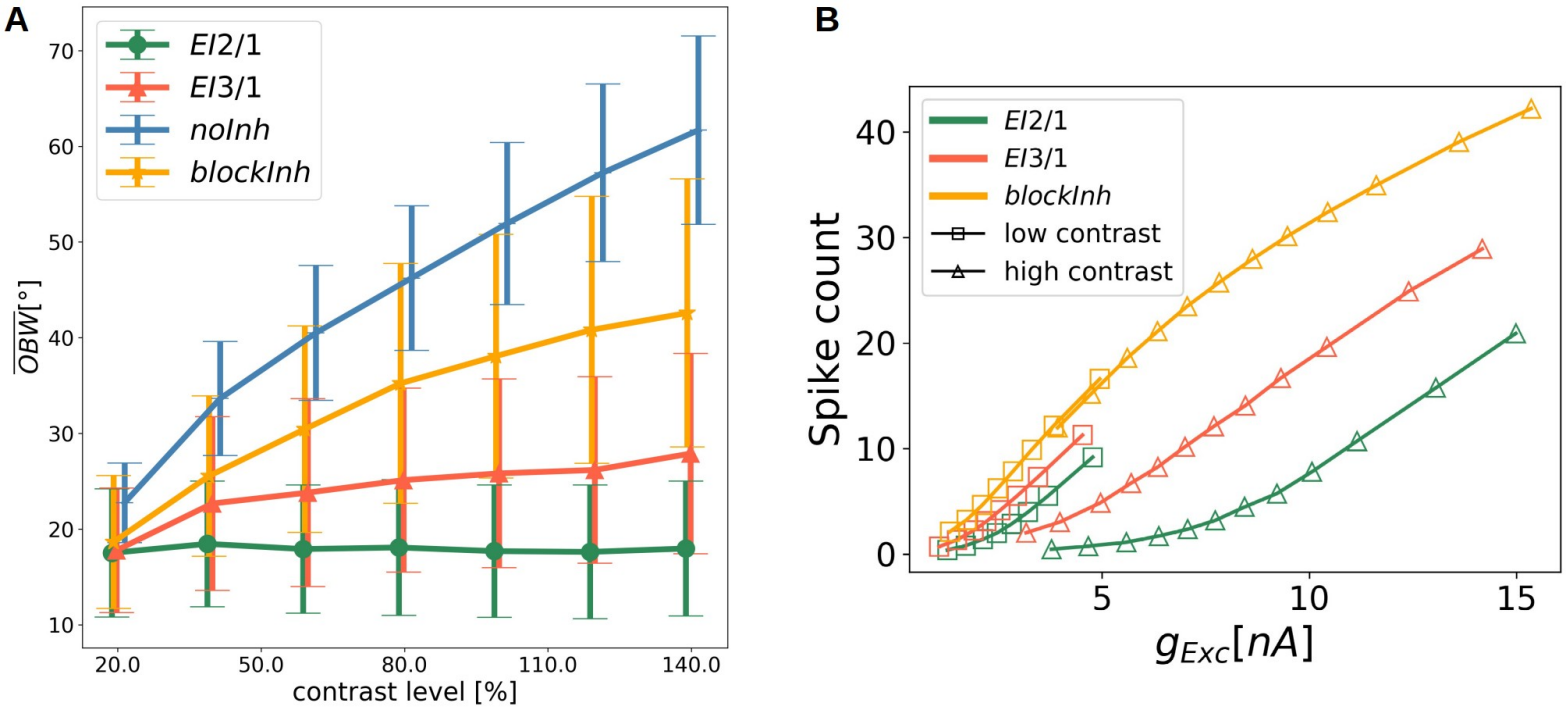
Response gain control by inhibition. **A** Mean OBW as a function of the contrast level in the input. Whiskers represent the standard deviation. Data from the *EI*2/1 model (green line), the model with all synapsed are from and to the inhibitory population are random and fixed (gray line), *EI*3/1 model (red line), and *noInh* model (blue line). **B** Spike count as a function of the excitatory input current for the *EI*2/1 model (green line), the *EI*3/1 model (red line) and the *blockInh* model (orange line). Data are taken from the sinusoidal tuning curve measurement, sorted by input current. Squares: Low input contrast. Triangles: High input contrast. Contrast-invariant tuning is only present in the *EI*2/1 model, while all other models show varying degrees of contrast-dependent widening of tuning curves.

Besides the sharpening of tuning curves, previous models suggest a role of inhibition in the invariance to input contrast changes [17, 45, 46]. However, those models assume hard-wired connectivity, and propose push-pull or anti-phase inhibition [45, 46]. Contrast-invariant V1 simple cells have been found in different mammals such as, cats [47, 48] or ferrets [49], based on sinusoidal gratings with different contrast strength. We use the same approach (see **Tuning curves and orientation selectivity**) to measure the tuning curves and calculated the averaged OBW over all excitatory cells for the different contrast levels (Fig 7A). Interestingly, the OBW is constant only for the *EI*2/1 model. For the model with weaker inhibition (*EI*3/1 model) and the model without inhibition (*noInh*), the OBW increases for higher input contrast values. Similarly, we observed a contrast-dependent increase in tuning width when inhibition was blocked after learning (*blockInh*). As it has been shown that random feedback inhibition is sufficient for the emergence of contrast invariant tuning curves [44], we omit data for the *fix ff inh* and *fix fb inh* models for clarity of display.

**Fig 8:**
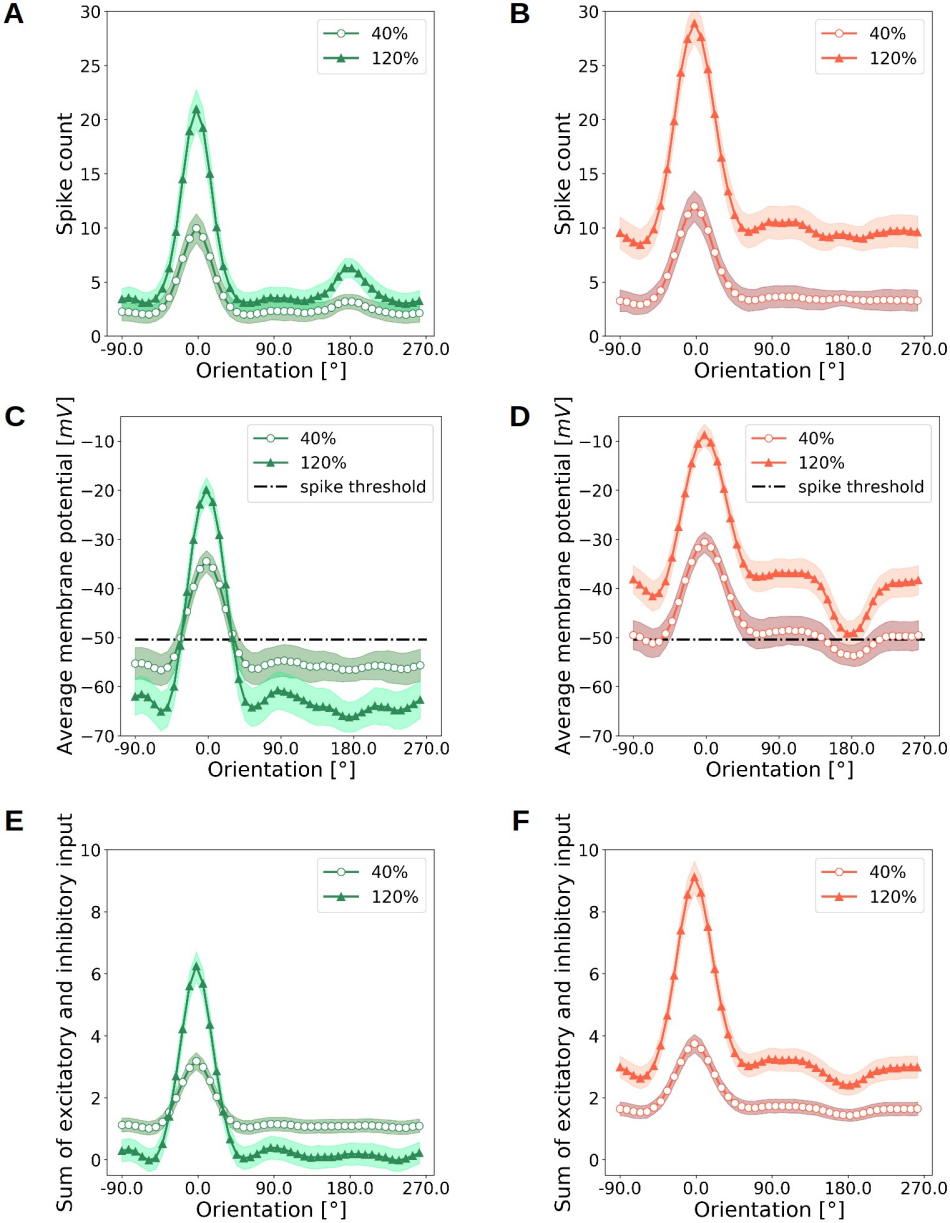
Emergence of contrast-invariant responses. **A** Average neural tuning curves for low and high contrast stimuli in the *EI*2/1 model, **B** and the *EI*3/1 model. **C** Average membrane potential (averaged across all neurons in the excitatory population) as a function of orientation and contrast level for the *EI*2/1 model, **D** and the *EI*3/1 model. **E** Sum of the excitatory and inhibitory input currents as a function of orientation and contrast level for the *EI*2/1 model, **F** and the *EI*3/1 model. In the *EI*3/1 model, high-contrast stimuli with non-preferred orientations are associated with very different dynamics than in the *EI*2/1 model: In the *EI*2/1 model, the sum of excitatory and inhibitory currents is near zero for non-preferred orientations at high contrast (**E**). In the *EI*3/1 model, the total synaptic current (**F**) remains large enough to elicit considerable membrane depolarization for non-preferred orientations at high contrast (**D**), reflected in elevated baseline activity and broader tuning (**B**).

To understand how the strength of inhibition affects contrast tuning curves, we compared the *EI*2/1 with the *EI*3/1 model with regard to their spike count, average membrane potential, and the average of the summed synaptic input current, for different contrast levels. At any contrast level, the activity of neurons in the *EI*2/1 model remains strongly suppressed at non-preferred orientations and increases around the preferred orientation (Fig 8A). By contrast, the *EI*3/1 model shows increased activity for high input contrast at all orientations (Fig 8B). This results in increased OBW values for higher input contrast (see also S9 Fig for normalized spike counts). Interestingly, for the non-preferred orientation, the average membrane potential the *EI*2/1 model is less hyperpolarized for lower contrast than for higher contrast. For higher contrast, the average membrane potential increases at the preferred orientation and is substantially stronger than for lower contrast. Both curves intersect around −50*mV*, close to the resting state spiking threshold (−50.4*mV*) (Fig 8C). This can be explained with the average input current: At higher contrast levels and non-preferred orientations, the feedback inhibitory current increases more strongly than the excitatory current and nearly compensates it (Fig 8E and S3(A) Fig), providing hyperpolarization of the membrane potential. This compensation of excitation decreases around the preferred stimulus, where the membrane potential exceeds the spiking threshold. In comparison, the membrane potential for the *EI*3/1 model increases proportionally with the total input current caused by higher input contrast (Fig 8D and 8F and S3(B) Fig). This suggests that the contrast-invariant tuning of the *EI*2/1 model depends on an appropriate balance between excitation and inhibition.

Based on the observation of contrast invariant tuning curves, we conclude that feedback inhibition modulates the neuronal gain controlled by input orientation and contrast. Fig 7B shows the average response gain for the excitatory population, averaged across the whole population, and sorted by the input current(see **Neuronal gain curves** for more details). We show the response gain curves for low and high contrast stimuli. For the model with blocked inhibition (*blockInh*), the gain curve is unaffected by contrast and follows the activation function defined by the neuron model. The firing rate to input ratios of neurons in the *EI*2/1 model are strongly reduced relative to the *blockInh* model, but this gain modulation is contrast-dependent, as the highest reduction of firing rates is observed for high contrast. This shows that the effect of inhibition on the neuronal gain function not only depends on the amount of excitatory input, but also on the stimulus orientation and contrast strength.

### Sparseness is increased by both, inhibition and tuning diversity

**Fig 9:**
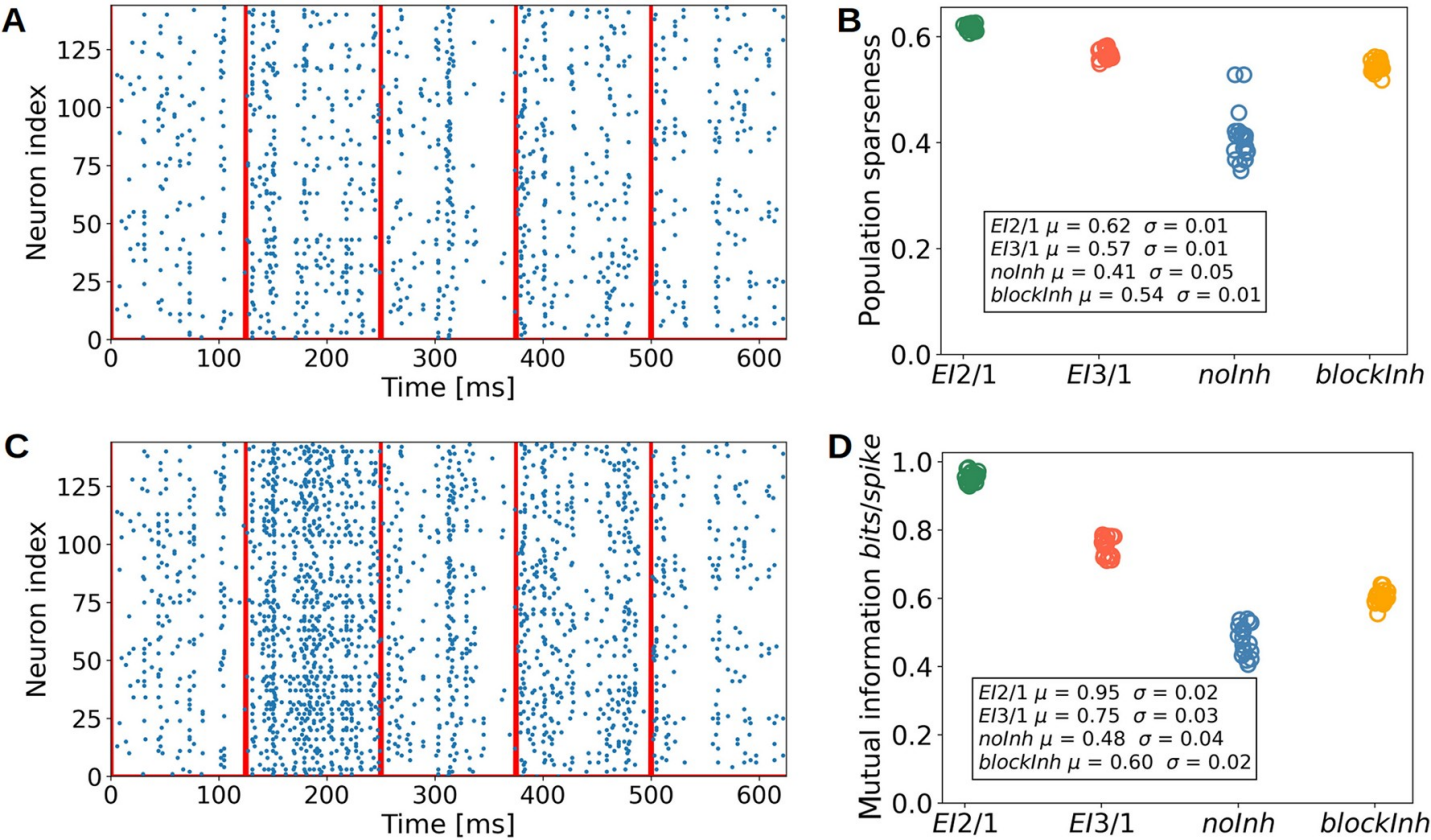
Sparse and efficient input representations through inhibitory processing. **A** Raster plot of the excitatory population for the *EI*2/1 model, same for the *blockInh* model **C**. Spikes are recorded on the same five natural image patches. The red lines show the stimulus onset. **B** Population sparseness for the *EI*2/1, the *blockInh*, and the *noInh* model, averaged across 10.000 natural scene patches. Higher value represent a higher sparseness of population activity. **D** Mutual information in *bits*/*spike* for the same three models as in **B**. **B** and **D** show data from 20 independent simulations per model configuration. Note the more synchronous population activity in the *noInh* model (**C**), associated with reduced sparseness (**B**) and lower information content (**D**). While blocking inhibition post-learning in the *blockInh* model decreases sparseness only moderately, it considerably reduces the information per spike.

As we observed that inhibitory processing led to an increase in the selectivity to artificial stimuli, we asked whether inhibition contributed to a sparser population code for natural images. We first compared the overall spiking behavior based on raster plots of network responses to five example image patches, for the *EI*2/1 (Fig 9A) and the *blockInh* model (Fig 9C). The model with active inhibition showed sparser firing and a less synchronous spiking behavior than the model with blocked inhibition. Second, to quantify this effect, we measured the population sparseness for all model configurations, based on the responses to 10.000 natural image patches (Fig 9B). The highest sparseness value (0.62) was observed in the *EI*2/1 model, 0.54 for the *blockInh* model and the lowest sparseness value (0.43) in the *noInh* model. Interestingly, the development of a higher diversity of receptive fields had a stronger influence on the population sparseness than inhibitory processing: Sparseness values differed more strongly between the model configurations without inhibition, the *noInh* and *blockInh* model, than between the *EI*2/1 and its blocked counterpart, which share the same feed-forward receptive fields.

### Metabolic efficiency benefits from strong feedback inhibition

The efficiency of information transmission (such as the numbers of spikes to represent specific input stimuli and the amount of information transmitted via a spike), or metabolic efficiency, is associated with the observed increase of the population sparseness [50]. To quantify the metabolic efficiency, we calculated the mutual information between input and response (see **Mutual information**). This analysis revealed a strong impact of inhibition on transmission efficiency (Fig 9D), normalized by spike count. The *EI*2/1 model shows the highest amount of information per spike (0.96 *bits*/*spike*). A lower inhibition strength in the *EI*3/1 model leads to a lower transmission efficiency (0.77 *bits*/*spike*). Both models without inhibition were associated with the least efficient population coding, with a lower value for the *blockInh* model, caused by a more diverse receptive field structure. To analyze further how the increase in information transmission was achieved, we calculated the discriminability index *d*′ on 500 randomly chosen natural scene patches to quantify the trial-to-trial fluctuation. We observed that higher *d*′ values were associated to both, high tuning diversity and the presence of inhibition(see S6 Fig). The improvement in discriminability is likely caused by a reduction of unspecific activity by inhibition, associated with more reliable stimulus representations, as observed in cat V1 [51] and mouse V1 [52]. In summary, our results show that the inhibitory processes in our models suppress redundant spikes which convey little information about the current stimulus [53].

Metabolic efficiency has also previously been linked to a minimum wiring principle [54] between neurons or cortical areas [54, 55]. While it would be interesting to explore effects of structural plasticity on metabolic efficiency, we here focused on the effects of inhibition.

### Input reconstruction benefits from plastic inhibition

**Fig 10:**
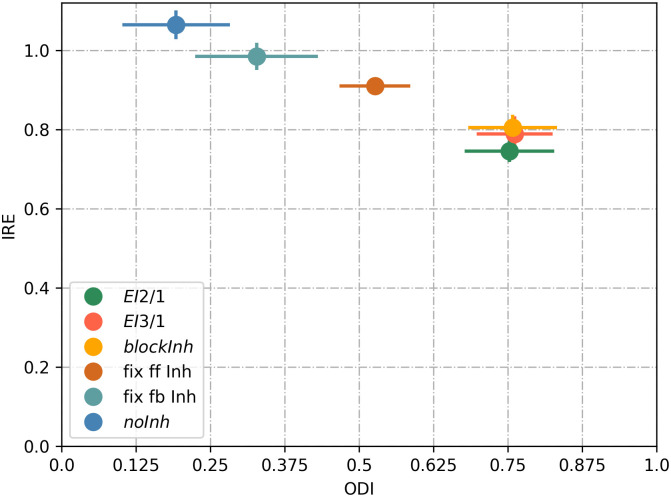
Plastic inhibition during learning improves input encoding quality via higher orientation diversity. Image reconstruction error (IRE) as a function of the orientation diversity index (ODI), for the *EI*2/1 model (green dot),the *EI*3/1 model (red dot), the *blockInh* model (orange dot), model with fixed feed-forward inhibition (brown dot), model with fixed feedback inhibition (light blue dot), and the *noInh* model (dark blue dots). IRE is calculated as the mean-square error between input image and the reconstruction. A better reconstruction is represented by smaller values for the IRE and is associated with a higher orientation diversity (presented by higher ODI values). Data shown from 20 independent simulations per model configuration.

We assume that a diversity of receptive fields, which encode the relevant input features, is crucial to provide an input representation without loss. To measure the quality of the input representation and to compare our model with existing sparse coding models, in terms of stimulus encoding, we calculated the image reconstruction error (IRE), which measures the mean-square error between the input image and its reconstruction obtained by linear decoding (see **Image reconstruction error**). We plot the IRE as a function of the receptive field diversity, measured by the orientation diversity index (*ODI*) as described previously (see **Orientation diversity**). The *EI*2/1 model with active and plastic inhibition during learning showed the lowest reconstruction error value (0.74), with a high ODI value (0.75) (Fig 10). By contrast, we observed a substantially smaller ODI value if there is no inhibition (*noInh* model) at all during learning (0.19), resulting in a higher reconstruction error (1.06). When the inhibition was blocked in the *EI*2/1 model after learning, the IRE shows a slight increase to a value of 0.79 (*blockInh* model). For the *EI*3/1 model we observed a similar IRE of 0.75 and a similar ODI value (0.76), indicating that the strength of inhibition during learning did not influence the emergence of receptive field diversity nor the input encoding quality.

If the feed-forward input to the inhibitory population is random and fixed during learning (*fix ff inh* model), the receptive fields of the excitatory population are less diverse, and the reconstruction error increases (0.91). A fixed inhibitory connection to the excitatory population (*fix fb inh* model) leads to a slightly higher reconstruction error (0.97) and a less diverse receptive field orientations (ODI of 0.33). This demonstrates that the plasticity of both the inhibitory feedback connections and the excitatory feed-forward connections to the inhibitory population leads to a better input representation, as a consequence of a higher receptive field diversity. Using fixed inhibitory feed-forward and feedback connections lead to a similar result then having only fixed feedback inhibitory connections (see S1 Fig).

To verify that the influence of plastic inhibition is the cause for a more receptive field diversity, and not a mechanisms of the chosen excitatory learning rule, we replaced the learning rule from [20] with the triplet STDP learning rule from [56]. We add a spike-traced based homeostatic mechanism (as suggest in [56]) to realize receptive field learning and tested fixed feed-forward, fixed feedback and non-plastic inhibition in the same way as for our original model. We observed the same reduction of orientation diversity with an increase in the IRE by fixed feed-forward and/or feedback inhibition (see S14 Fig). Together, these results indicate that the diversity of receptive fields contributes to the average reconstruction accuracy. Further, after learning, the effect of active inhibition on the encoding quality is negligible. This is important, as inhibition is essential for receptive field diversity, but it may contribute to a loss of information if the neural code becomes too sparse by the suppression of too many feature-coding neurons [35]. This is crucial for a robust input representation, where a very sparse representation (or local code) is less robust against noise [50]. We already showed in two previous studies with similar neural networks, how inhibition can increase the robustness against the loss of information in the input (what can be understood as noise) [57, 58]. Additional, we measured the resulting image reconstruction error with white noise added on a natural scene and observe a higher robustness against noise in models with plastic inhibition (see S15 Fig).

## Discussion

Our model suggests that a single underlying mechanism—the interaction of excitatory and inhibitory plasticity—can explain the stable emergence of reliable and efficient input encoding. We have shown that in particular, the combination of plastic inhibitory feedback and plastic feed-forward inhibition has an influence on shaping the receptive fields. Our simulation results are supported by recent physiological findings that inhibitory plasticity influences the mode of operation of excitatory neurons (for example the excitability) [9, 10, 59, 60], or influences the occurrence of LTP and LTD [11, 59, 61].

Previous models based on STDP rules, which have demonstrated the emergence of V1 simple cells, made several simplifications in terms of the learning dynamics [8, 12, 13], or considered plasticity only for a subset of projections [14, 15]. These assumptions make it difficult to investigate the influence of plastic feed-forward and feedback inhibition on network dynamics and input encoding. For example, the observation of response decorrelation is a direct consequence of the chosen learning mechanism [8, 13]. Other learning rules have been designed to optimize the mutual information between input and output [12]. This suggests that a more detailed model of V1 circuit development is necessary to understand the dynamics between excitation and inhibition during learning. To advance our understanding of this process, we investigated a spiking network model of V1 simple cell development, based on two phenomenological learning rules implemented at all synaptic projections.

### Feed-forward and feedback inhibitory plasticity improves orientation diversity and representational efficiency

Our results show that plastic inhibitory feedback as well as plastic feed-forward inhibition influence the development of V1 simple cells, lead to a higher orientation diversity, and improve representational efficiency. Inhibitory plasticity has been reported in numerous physiological studies [4, 9, 10, 62, 63, 64]. Previous model studies suggest a role for inhibitory plasticity in controlling the balance between excitation and inhibition [21, 65], or in enabling stability in recurrent networks [65, 66]. However, there is ongoing discussion about the necessity and role of inhibitory plasticity during learning a functional sensory code book [59, 66, 67], and this issue has received limited attention in model studies so far.

In a model based on a combination of STDP and inhibitory STDP learning rules, Litwin-Kumar and Doiron [65] showed that inhibitory plasticity is necessary for stable learning in a network with recurrent excitatory connections. Their study used a generic cortical network receiving non-plastic input from a set of 20 artificially stimuli, which in turn resulted in the formation of 20 assemblies representing the input stimuli. They emphasized that inhibitory plasticity acted to equilibrate firing rates in the network, such that different assemblies (each coding for one stimulus) received different amounts of inhibition, preventing dominant activity of single assemblies. Our results of a feature-specific strength of inhibition generalize their finding of firing rate heterogeneity induced by iSTDP from an “assembly code”, in which different stimuli rarely overlap, to the quasi-continuous space of natural visual stimuli. This supports the necessity of the interaction of inhibitory and excitatory plasticity during the development of the visual cortex.

### Emergence of a self-organized balance of excitation and inhibition

Based on natural scene stimuli, we observed in our model that the inhibitory input current to a neuron is proportional to the excitatory input, when the currents are averaged across the duration of a stimulus. However, as we did not observe an equal strength between these currents, excitation is dominant in our network. This indicates a detailed and loose balance (for definition see, Hennequin et al. (2017) [68]) between excitation and inhibition in our network. While a detailed balance has been reported in rat auditory cortex [69], it is still under discussion if a more loose or tight balance exists in the primary visual cortex of higher mammals [70]. Recent model studies suggest a tight balance between inhibition and excitation [71] or rather an inhibitory dominated network for stable learning in a network with recurrent excitatory synapses [14, 15, 65]. However, most of these models investigate excitation-inhibition balance in a single-neuron setup [71], or set a subset of synaptic connections fixed [14, 15, 65]. Interestingly, we observed that the ratio between excitation and inhibition changes in our network for different contrast levels of sinusoidal grating stimuli, up to a 1 : 1 balance for the highest contrast level for the *EI*2/1 model. This shows that the balance between excitation and inhibition is input-specific.

### Inhibition implements a gain control mechanism and shapes tuning curves

Previous physiological studies found that parvalbumin-expressing (PV) interneurons have a divisive impact on the gain function of pyramidal neurons in the visual cortex, to implement a contrast gain control mechanism [72, 73, 74]. In our model we observed that the ratio between excitatory and inhibitory currents influences the response of the neuron towards its input. Consequently, feedback inhibition implements a gain control mechanism for the excitatory neurons.

Savin et al. [12] proposed a rapid intrinsic plasticity mechanism to adapt the neuronal gain function to optimize the information transmission between input stimuli and neuronal output. They suggested that the emergence of V1 simple cell receptive fields depends on the interplay between the adaption of the neuronal gain function and the synaptic plasticity [12]. By contrast, in our network, changes in neuronal gain curves are caused by feedback inhibition, which adapts at the fast time scale of synaptic plasticity to maintain a given target rate.

In our model, when blocking inhibition after learning, we observed an increase not only in the baseline activity, but also in the orientation bandwidth (OBW). This demonstrates a sharpening of tuning curves by inhibition, similar to the observation in [75], where inhibitory synapses in cat primary visual cortex were blocked with gabazine. Interestingly, PV cells seem not to affect the sharpening of tuning curves [72, 73], whereas somatostatin-expressing neurons (SOM) sharpen neuronal responses [73]. This demonstrates the influences of the different inhibitory neuron types [16], which must be taken into account in future models.

### Shift in the E/I balance leads to the spontaneous emergence of contrast invariant tuning curves

As a consequence of the contrast gain mechanism by inhibition, our model shows the spontaneous emergence of contrast invariant orientation tuning [45, 47, 48]. Early modeling studies have proposed feed-forward inhibition to implement a push-pull inhibitory mechanism for the emergence of contrast-invariant tuning curves [45, 46]. Despite the fact that our network contains feed-forward inhibition, we did not observe a push-pull inhibitory effect, in other words, anti-correlation of excitation and inhibition [76]. To be more specific, a direct comparison of the excitatory and inhibitory input current for the contrast invariance task shows a simultaneous increase and decrease of excitation and inhibition, caused by the detailed balance in our network (see S7 Fig). We have observed that for the *EI*2/1 model, inhibitory input currents increase more rapidly than excitatory currents at higher contrast levels and non-preferred orientations. This results in a shift from a two-to-one ratio of excitation to inhibition to a one-to-one ratio between excitation and inhibition, and implements a contrast-dependent modulation of the neuron’s gain curve. In contrast to that, we observed for the *EI*3/1 model a proportional growth of the excitatory and inhibitory input currents for higher input contrast (see S8 Fig), leading to an increase of the OBW. This shows that the emergence of contrast-invariant tuning curves is an inherent effect of the ratio between excitation and inhibition in our network, and suggests that contrast invariance emerge at a specific E/I ratio. A contrast-dependent shift in the balance between excitation and inhibition has been reported in the visual cortex of awake mice [77]. Although the influence of inhibition on the neuronal gain function for the emergence of contrast invariance is in line with previous assumptions [48, 78], recent studies have proposed that changes in the neuronal gain are caused by response variability in the afferent thalamic path [79, 80]. An alternative proposal holds that fixed unspecific inhibition leads to contrast invariance [44]. We confirmed this by shuffling all synaptic weight to and from the inhibitory population. In this condition, we observed contrast-invariant tuning (see S10 Fig). Our results extend these previous theories by showing that specific inhibition, as emerging through inhibitory plasticity and given sufficient inhibitory strength, is a sufficient condition for contrast invariance as well.

### Sparseness and metabolic efficiency benefit from E/I balance

We observed that in the *EI*2/1 model, the standard deviation of the membrane potential increases for non-preferred orientations. Together with the observed asynchronous spiking behavior, we conclude that the balance of inhibition and excitation leads to a more irregular spiking behavior. Previous work suggests that a more irregular activity and irregular membrane potential behavior is related to improved metabolic efficiency in terms of efficient input encoding [71]. Our observations agree with these findings, because the efficiency of information transmission in our network mainly benefits from the ratio between excitatory and inhibitory currents in the stable network.

An established approach in terms of input encoding efficiency is the concept of sparse coding [81, 82, 83]. However, in recent years, it has been discussed how the level of sparseness reported in physiological experiments is influenced by animal age and the level of anesthesia [84], and the benefit of highly sparse codes for information processing has been questioned [35, 50, 85]. Overall, the intermediate sparseness values observed in our model are in agreement with experimental findings [1, 84].

### Structured connectivity caused by inhibitory and excitatory plasticity

Previous physiological studies have shown that inhibitory interneurons are connected in a nonspecific manner to other cells in their surrounding [86]. However, recent studies observed that inhibitory PV cells develop strong connections to excitatory cells with similar orientations [60], and that neurons with similar preferred orientations have a higher probability for recurrent connections [34, 87].

We observed a similar connectivity pattern in our network, namely, the appearance of strong connectivity between co-tuned neurons. King et al. [13] also obtained a structured connectivity between co-tuned excitatory and inhibitory cells in a spiking network. While they achieved this goal by designing a suitable learning rule for the synaptic projections involving inhibitory neurons, we observed the appearance of strong connectivity as an emergent property of our model architecture based on detailed phenomenological rules.

### Stable learning despite limitations of simultaneous excitatory and inhibitory plasticity

Previous studies have mentioned the difficulty to achieve a certain level of inhibition in a network with inhibition and plastic excitatory synapses [68, 88]. We next discuss the behavior of the selected learning rules more in detail to show some of the difficulties during the interaction of excitatory and inhibitory plasticity, and discuss the limitations of our modeling approach.

For the excitatory learning rule, it has been shown in [20] that a lower input firing rate leads to bigger receptive fields, as a compensatory effect of the homeostatic mechanism. This mechanism is controlled by the long-term postsynaptic membrane potential in relation to a reference value and implements a local homeostatic mechanisms to influence the synaptic plasticity. If the membrane potential is too low, less long-term depression (LTD) in relation to long-term potentation (LTP) occurs, and the weights will increase. Otherwise, if the membrane potential is too high, a higher amount of LTD will occur to decrease the weights. Consequently, for a lower input firing rate, more weights will increase, saturating at their maximum, to achieve a specific postsynaptic activity.

The homeostatic mechanism of the inhibitory rule [21] strengthens the inhibition if the postsynaptic activity is too high, with respect to a target firing rate (*ρ*), or decreases the weight otherwise. In our network, the postsynaptic membrane potential is a result of the difference between the incoming excitatory and inhibitory current, such that a reduction in the membrane potential through inhibition is comparable to a reduction through less presynaptic spikes. The operation of both homeostatic mechanisms on the postsynaptic activity leads to a competition between weight changes at excitatory and at inhibitory synapses and should lead to bigger receptive fields, or, in the worst case, to a saturation of all synapses to their maximum value (see S2 and S3 Figs).

However, we observed the emergence of stable receptive fields and stable connections between the populations. Additionally, our results show a reduction in the mean activity, caused by inhibition, without causing bigger receptive fields. We assume that in contrast to a reduction in the input, what leads to a proportional reduction on the postsynaptic neuron, the inhibitory current leads to a more irregular, or fluctuating, behavior of the membrane potential [89]. To allow LTP at excitatory synapses, the membrane potential must be higher than *θ*_+_ (= −45.3*mV*), which is slightly above the steady-state spiking threshold ($V_{T_{rest}}=-50.4mV$). But if the membrane potential is hyperpolarized by inhibition, it falls below the LTP threshold: No LTP occurs, and the weights will not increase to the maximum. Additionally, we observed that the interplay of the excitatory and inhibitory rules are mainly influenced by the magnitude of learning rates. In particular, a higher excitatory or higher inhibitory learning rate led to the saturation of all synapses, as an effect of the competition between both homeostatic mechanisms. How fast the synaptic weight changes depends not only on the magnitude of learning rates, but also on the number of spikes, that is, the number of learning events. Therefore, the learning rates for the *noInh* model is smaller, to compensate the higher activity in the neuron populations. Finally, the competitive pressure between learning rules is controlled by the postsynaptic target activity in the inhibitory learning rule. Smaller values of *ρ* enhances the inhibitory pressure on the post-synaptic neuron to achieve a lower firing rate and can also lead to an unlimited growth of synaptic weights. This limited the amount of inhibition that can emerge in the network and did not allow a one-to-one balance between excitation and inhibition in our model, at least for natural scene stimuli. However, when presenting sinusoidal gratings of high contrast, E/I balance shifted towards a 1:1 ratio in the *EI*2/1 model, suggesting that this balance is stimulus-dependent.

Previous model studies reported that receptive fields can emerge without inhibition, by maintaining the post-synaptic activity over intrinsic plasticity [12], implementing a BCM-like behavior with a post-synaptic spike trace [56], or regulating the LTD-term [20]. As expected by the chosen learning rules, our simulations with the *noInh* model confirm the emergence of receptive fields without inhibition. Despite this, other model studies pointed out the role of local homeostatic mechanisms on the emergence of selective receptive fields [8, 90, 91] in networks with inhibition, or proposed that inhibition increases the diversity of receptive fields by implementing a competition between neurons [12, 13, 92]. In addition, our results show that plastic inhibition increases the receptive field diversity in comparison to fixed inhibition. By starting from unselective neurons, they develop a simple selectivity which pushes the correlation-based inhibitory influence to force a decorrelation between neurons and increase orientation divergence. This shows that inhibitory plasticity not only maintains the postsynaptic activity, but also implements a selective competition between neurons during a highly dynamical phase of development. Previous experimental studies mentioned different phases during the cortical maturation [93, 94, 95], discussed the role of inhibition for the beginning of a critical period [93, 94], or showing a temporal decrease of inhibition to enable synaptic plasticity [95]. One of the best studied examples of critical period in the visual cortex is the onset of ocular dominance (OD) plasticity [94, 95, 96]. It has been discussed earlier that inhibitory interneurons (especially PV+) are important for the regulation of OD plasticity [94, 95] and the strength of inhibition itself changes during this critical period [95, 97], like a rapid downregulation of inhibitory cell activity [95, 98]. Our study about the role of inhibition for learning provides an excellent starting point for studies that aim to look at different critical periods in development.

## Conclusion

To the best of our knowledge, our simulations are the first demonstration of the parallel emergence of fundamental properties of the primary visual cortex such as sparse coding, contrast invariant tuning curves and high accuracy input representation, in a spiking network with spike timing-dependent plasticity rules. A central finding of our study is that the emergence of representational efficiency (such as tuning diversity) requires plasticity at feed-forward and feedback inhibitory synapses. Further, the emergence of a high tuning diversity as a direct consequence of inhibitory plasticity provides a verifiable prediction, via pharmacological or genetic methods, which allow to suppress inhibitory plasticity during the development of V1 simple cells. Although previous research has shown that unspecific inhibition has an effect on the gain-function of the excitatory cells, to improve the metabolic efficiency [99] or to cause contrast invariance [44], our results demonstrate that the E/I ratio emerging from learning increases the metabolic efficiency (in terms of bits per spike) in our network. This emphasizes the role of inhibition in the shaping of neuronal responses [5, 43, 66] and in the development of reliable and efficient input encoding.

## Materials and methods

The first part of this section describes the network architecture including the neuron model and learning rules. The model has been implemented in Python 3.6, using the ANNarchy simulator [100], with a simulation time step of *dt* = 1*ms* (Euler integration). The neuronal simulator is available from https://bitbucket.org/annarchy/annarchy. The implementation of the adaptive exponential integrate-and-fire neuron model and the voltage-based triplet STDP learning rule proposed in [20] based mainly on the re-implementation in [101].

### Network architecture

Our network model, which is inspired by the primary visual cortex and its inputs from LGN, consists of three populations of spiking neurons (Fig 1A): An input layer representing LGN, and excitatory and inhibitory populations of V1, each receiving feed-forward inputs from LGN. The V1 populations are mutually interconnected via excitatory or inhibitory synapses, respectively. The circuit therefore implements both feed-forward and feedback inhibition, in agreement with anatomical findings [5]. Inhibitory interneurons receive additional recurrent inhibitory connections. All projections follow an all-to-all connectivity pattern, excluding self inhibitory feedback connections.

The LGN layer consists of 288 neurons showing Poisson activity and is split into ON- and OFF-subpopulations. For the V1 excitatory population (144 neurons) and the inhibitory population (36 neurons), we used adaptive exponential integrate-and-fire neurons (see **Adaptive exponential integrate-and-fire neurons in V1**). The size of the inhibitory population was chosen to match the 4:1 ratio between excitatory and inhibitory neurons found in visual and striate cortex [16, 18, 19]. Researchers reported a much higher volume for the primary visual cortex than the LGN [102], what suggests a much higher number of neurons. We verified tat the mere size of V1 in our model does not influence our conclusions, by increasing the number of excitatory and inhibitory cells by the factor of 2 and 10 using a sparse connectivity between excitatory and inhibitory cells to guaranty a similar E/I balance than for the *EI*2/1 model. We measured the input reconstruction error and the orientation bandwidth on different contrast levels and did not observe a high difference in comparison to the *EI*2/1 model (see S12 Fig).

All synaptic connections within our model are plastic and were randomly initialized. They change their weight based on either the voltage-based STDP-rule proposed by Clopath et al. [20] (excitatory connections) or the symmetric iSTDP-rule proposed by Vogels et al. [21] (inhibitory connections; Sec. **Synaptic plasticity**).

Although networks of the visual cortex have lateral excitatory connections [86, 87, 103, 104] as also discussed in different model studies [14, 15, 65], we did not insert plastic lateral excitatory connections in our model, as our model is already highly adaptive and further excitatory connections may complicate the required set of learning rules. However, to observe the influence of lateral excitation, we inserted fixed excitatory connections with a connection probability of 0.2 between the excitatory neurons and initialized the weight with an uniform distribution. Despite the number of unstable learning approaches increased (see S13 Fig), we did not observe a significant influence of the recurrent connections, by measuring the IRE and OBW.

### Network input

As network input, we used whitened patches from natural scenes [6, 105]. Each patch was chosen randomly, with a size of 12 by 12 by 2 pixels [35]. The third dimension corresponds to the responses of ON- and OFF-cells. To avoid negative firing rates, we mapped positive pixel values to the ON-plane, and the absolute value of negative pixels to the OFF-plane. Every patch was normalized with the maximum absolute value of the corresponding natural scene. The firing rate of each Poisson neuron represents the brightness value of the input pixels. The firing rate associated to the (rarely occurring) maximum pixel value was set to 125*Hz*. We stimulated the network with 400.000 patches during training, with a presentation time of 125*ms* per patch, corresponding to around 14*h* of simulated time. To avoid any orientation bias in the input, the patch was flipped around the vertical or horizontal axis independently with 50% probability [20].

### Poisson neuron model in LGN

For modeling convenience, we generated Poisson activity in LGN neurons by injecting brief voltage pulses, generated by a Poisson process, into a binary spiking neuron model, such that each voltage pulse input triggered a spike. This simplified the numerical calculation of a spike trace required for the learning rule, while preserving the precise timing of spikes drawn from a Poisson process.

The spike trace ${\bar{x}}_{i}$ is updated whenever the presynaptic neuron *i* spikes, and decays exponentially: *X*_*i*_(*t*) = 1 if a spike is present at time *t*, and *X*_*i*_(*t*) = 0 otherwise.

$$\begin{array}{c} \frac{du}{dt}=I_{Poisson} \end{array}$$ (1)

$$\begin{array}{c} \tau_{x}\frac{d{\bar{x}}_{i}}{dt}=-{\bar{x}}_{i}+X_{i} \end{array}$$ (2)

### Adaptive exponential integrate-and-fire neurons in V1

For the neurons in the V1 excitatory and inhibitory layer, we used a variant of the adaptive exponential integrate-and-fire model as described in [20]. In this model, the membrane potential *u* is influenced by the following additional dynamical variables: An adaptive spike threshold, *V*_*T*_, a hyperpolarizing adaptation current, *w*_*ad*_, and a depolarizing afterpotential, *z*. Excitatory and inhibitory synaptic currents are denoted by *I*_*exc*_ and *I*_*inh*_. For an explanation of constant parameter values as used in [20], see Table 1.

**Table 1:** Parameters for the neuron model and excitatory synapses.

Global parameter values			
Parameter (values from Clopath et al. [20])	Value	Parameter	Value
*C*, membrane capacitance	281*pF*	*τ*_*z*_, spike current time constant	40*ms*
*g*_*L*_, leak conductance	30*nS*	$\tau_{V_{T}}$ , spike threshold time const.	50*ms*
*E*_*L*_, resting potential	−70.6*mV*	*τ*_*x*_, spike trace time constant	15*ms*
Δ_*T*_, slope factor	2*mV*	*τ*_*wad*_, adaption time constant	144*ms*
$V_{T_{rest}}$ , spike threshold at rest	−50.4*mV*	*I*_*sp*_, spike current after spike	400*pA*
$V_{T_{max}}$ , spike threshold after spike	30.4*mV*	*a*, subthreshold adaptation	4*nS*
$w_{min}^{e}$ , min. excitatory weight	0.0	*b*, spike-triggered adaption	0.805*pA*
*τ*_−_, time constant for ${\bar{u}}_{-}$	10.0*ms*	*τ*_+_, time constant for ${\bar{u}}_{+}$	7.0*ms*
*θ*_−_, plasticity threshold	−70.6*mV*	*θ*_+_, plasticity threshold (LTP)	−45.3*mV*
Parameter (added)	Value	Parameter	Value
$\tau_{I_{exc}}$ , excitatory input time const.	1.0*ms*	$\tau_{I_{inh}}$ , inhibitory input time const.	10.0*ms*
Projection-specific parameters			
Parameter (custom values)	*LGNtoE*	*LGNtoI*	*EtoI*
$\tau_{\bar{\bar{u}}}$	750*ms*	750*ms*	750*ms*
$w_{max}^{e}$	5.0	3.0	1.0
*w*_*init*_ (bounds of random	[0.015, 2.0]	[0.0175, 2.15]	[0.0175, 0.25]
uniform distribution)			
*A*_*LTP*_ (*EI*2/1, *EI*3/1)	1.35 × 10^−4^	5.4 × 10^−5^	1.2 × 10^−5^
*A*_*LTD*_ (*EI*2/1, *EI*3/1)	1.05 × 10^−4^	4.2 × 10^−5^	1.4 × 10^−5^
*A*_*LTP*_ (*noInh*)	7.2 × 10^−5^	n/a	n/a
*A*_*LTD*_ (*noInh*)	5.6 × 10^−5^	n/a	n/a
${\bar{\bar{u}}}_{ref}$	60.0*mV*^2^	55.0*mV*^2^	55.0*mV*^2^

Note that for the *noInh* model, learning rates were reduced to compensate for the increased firing rates in the absence of inhibition.

The full equation for the membrane potential is

$$\begin{array}{c} C\frac{du}{dt}=-g_{L}\left(u-E_{L}\right)+g_{L}\Delta_{T}e^{\frac{u-V_{T}}{\Delta_{T}}}-w_{ad}+z+I_{exc}-I_{inh} \end{array}$$ (3)

As the triplet voltage STDP rule is sensitive to the precise time course of the membrane voltage, including the upswing during a spike, the magnitude of weight changes depends on the implementation details of the after-spike reset. To avoid long simulation times associated with smaller time steps, we opted for the following simplified treatment of the spike waveform which reproduced the results reported by Clopath et al. [20]: Whenever the membrane potential *u* exceeded the spike threshold, *u* was held at a constant value of 29*mV* for 2*ms*, and then reset to the resting potential *E*_*L*_. We obtained highly similar results from an alternative implementation, in which the after-spike reset was immediately applied when the spike threshold was crossed, with an additional update of the voltage traces by the amount expected from a 2*ms*-long spike.

The reset value for the spike threshold is $V_{T_{max}}$, with exponential decay towards the resting value $V_{T_{rest}}$, with a time constant $\tau_{V_{T}}$ (Eq 4):

$$\begin{array}{c} \tau_{V_{T}}\frac{dV_{T}}{dt}=-\left(V_{T}-V_{T_{rest}}\right) \end{array}$$ (4)

The afterpotential *z* has a reset value of *I*_*sp*_ and decays to zero (Eq 5). Further, the variable *w*_*ad*_ is incremented by the value *b* and decays exponentially (Eq 6).

$$\begin{array}{c} \tau_{z}\frac{dz}{dt}=-z \end{array}$$ (5)

$$\begin{array}{c} \tau_{w_{ad}}\frac{dw_{ad}}{dt}=a\left(u-E_{L}\right)-w_{ad} \end{array}$$ (6)

The model proposed by Clopath et al. [20] assumed excitatory synaptic input in the form of voltage pulses. For modeling convenience, we approximated this setting by current-based excitatory synapses with a short time constant of 1*ms*. Inhibitory synaptic currents decayed with a slower time constant of 10*ms*. Both synaptic currents are incremented by the sum of synaptic weights of those presynaptic neurons which spiked in the previous time step:

$$\begin{array}{c} \begin{array}{c} \tau_{I_{exc}}\frac{dI_{exc}}{dt}=-I_{exc}+w_{i}^{exc}\sum_{i\in Exc}\delta \left(t-t_{i}^{{}^{\prime }}\right)\\ \tau_{I_{inh}}\frac{dI_{inh}}{dt}=-I_{inh}+w_{j}^{inh}\sum_{j\in Inh}\delta \left(t-t_{j}^{{}^{\prime }}\right) \end{array} \end{array}$$ (7)

where $t_{i}^{{}^{\prime }}$ denotes the spike time of presynaptic neuron *i*, and *δ* is the indicator function with *δ*(0) = 1.

### Synaptic plasticity

#### Voltage-based triplet STDP at excitatory synapses

Plasticity at excitatory connections (LGN to Exc., LGN to Inh. and Exc. to Inh.) follows the voltage-based triplet STDP rule proposed by Clopath et al. [20]. We here repeat the essential features of this plasticity model. The neuronal and synaptic variables describing the development of the weight from a presynaptic neuron with index *i* onto a given postsynaptic neuron are: *X*_*i*_, the presence of a presynaptic spike; ${\bar{x}}_{i}$, the presynaptic spike trace (Eq 2); *u*, the postsynaptic neuron’s membrane potential; and two running averages of the membrane potential, ${\bar{u}}_{+}$ and ${\bar{u}}_{-}$, defined as follows:

$$\begin{array}{c} \tau_{+}\frac{d{\bar{u}}_{+}}{dt}=-{\bar{u}}_{+}+u, \end{array}$$ (8)

where ${\bar{u}}_{-}$ is defined analogously, with the time constant *τ*_−_. In addition, the learning rule includes a homoeostatic term, $\bar{\bar{u}}$, which regulates the relative strength of LTD versus LTP, and which measures the mean postsynaptic depolarization on a slower time scale:

$$\begin{array}{c} \tau_{\bar{\bar{u}}}\frac{d\bar{\bar{u}}}{dt}={\left[{\left(u-E_{L}\right)}^{+}\right]}^{2}-\bar{\bar{u}} \end{array}$$ (9)

Here, *x*^+^ = max(*x*, 0) denotes top-half rectification.

The full learning rule is given as the sum of the LTP term and the LTD term:

$$\begin{array}{c} \frac{dw_{i}}{dt}=A_{LTP}\;{\bar{x}}_{i}{\left(u-\theta_{+}\right)}^{+}{\left({\bar{u}}_{+}-\theta_{-}\right)}^{+}-A_{LTD}\frac{\bar{\bar{u}}}{u_{ref}}X_{i}{\left({\bar{u}}_{-}-\theta_{-}\right)}^{+} \end{array}$$ (10)

where *A*_*LTP*_ and *A*_*LTD*_ are the learning rates for LTP and LTD, *θ*_+_ and *θ*_−_ are threshold parameters, and *u*_*ref*_ is a homeostatic parameter which controls the postsynaptic target firing rate. Clopath et al. (2010) [20] have shown that this learning rule results in BCM-like learning dynamics [106], in which a sliding metaplasticity threshold leads to the development of selectivity.

Following Clopath et al. [20], for the LGN efferent connections, we equalized the norm of the OFF weights to the norm of the ON weights every 20*s*. The weight development is limited by the hard bounds $w_{min}^{e}$ and $w_{max}^{e}$. Parameter values for the excitatory synapses can be found in Table 1.

#### Homeostatic inhibitory plasticity

Previous biological studies have observed spike timing-dependent plasticity of inhibitory synapses which differs from the well-known asymmetric STDP window [64, 107]. We therefore chose to implement the phenomenologically motivated, symmetric inhibitory STDP (iSTDP) rule proposed by Vogels et al. [21] at all inhibitory synapses (Eq 11):

$$\begin{array}{c} w\left(t+dt\right)=\{\begin{array}{cc} w\left(t\right)+\eta \left({\bar{x}}_{post}-\rho \right) & \text{if}\;t=t_{pre}\;\text{(presynaptic}\;\text{spike)}\\ w\left(t\right)+\eta {\bar{x}}_{pre} & \text{if}\;t=t_{post}\;\text{(postsynaptic}\;\text{spike)} \end{array} \end{array}$$ (11)

Here, *η* is the learning rate, and *ρ* is a constant which controls the amount of LTD relative to LTP. Further, [21] have shown that this learning rule has a homeostatic effect, and the parameter *ρ* controls the postsynaptic target firing rate. The variables ${\bar{x}}_{pre}$ and ${\bar{x}}_{post}$ are spike traces for the pre- and postsynaptic neurons, defined in analogy to Eq 2, with time constants *τ*_*pre*_ and *τ*_*post*_. In this plasticity rule, near-coincident pre- and post-synaptic spiking causes potentiation of weights, irrespective of their temporal order. By contrast, isolated pre- or postsynaptic spikes cause depression of weights. As for the excitatory learning rule, weights are bounded by $w_{min}^{i}$ and $w_{max}^{i}$. For parameter values, see Table 2.

**Table 2:** Parameters for inhibitory synapses.

	ItoE and ItoI	ItoE	ItoI
*τ*_*post*_	10.0*ms*		
*τ*_*pre*_	10.0*ms*		
*w*^*i*^ initial	0.0		
$w_{min}^{i}$	0.0		
$w_{max}^{i}$		0.7	0.5
*η*		10^−5^	10^−5^
*ρ* (*EI*3/1)		0.7	0.6
*ρ* (*EI*2/1)		0.4	0.6

#### Choice of parameter configurations

As our main goal is to determine the influence of inhibitory strength both on the formation of selectivity and on the dynamics of stimulus coding, we simulated our network using different parameter and network configurations. First, we used the above presented network, where the strength of the inhibitory feedback is controlled by the homeostatic parameter *ρ*. With *ρ* = 0.4 for the feedback inhibitory synapses, we achieved a ratio of excitation to inhibition (E/I-ratio) of approximately 2 : 1 on patches of natural scenes (abbreviated as *EI*2/1). On one hand, a lower *ρ* would strengthen the inhibitory feedback, but caused unstable behavior during learning. On the other hand, a higher *ρ* would weaken the inhibitory feedback of the model. Because of this, we were unable to achieve a 1 : 1 E/I ratio for natural scene patches. With *ρ* = 0.7 we achieve a E/I-ratio of approximately 3 : 1 on natural scene input (abbreviated as *EI*3/1), this led to similar but weaker characteristics for most of the experiments (Fig 1B).

Second, we simulated a purely excitatory feed-forward network without any inhibitory activity (abbreviated as *noInh*), as the learning rule proposed by Clopath et al. [20] is capable of learning distinct shapes of receptive fields given different initial weights.

Further, to control for the dynamical effects of inhibition in the steady state following receptive field development, we simulated the effects of deactivating the inhibitory synaptic transmission in the *EI*2/1 model after learning (abbreviated as *blockInh*). All three model variations are based on the same network architecture, consisting of the same number of neurons in each population and the same number of synapses, except that inhibitory weights differ in their strength or are deactivated. The different parameters for learning the models are shown in Table 1. We took the parameters for the adaptive integrate and fire neuron from Clopath et al. [20]. Based on the original parameter mentioned in Clopath et al. [20] and Vogels et al. [21], the parameters for both learning rules were found empirically to enable a stable emergence of receptive fields in multiple runs, initialized with different weight values (see S11 Fig).

To test the stability and the reproducibility of our results, we performed 20 runs of each model with randomly initialized synaptic weights.

To evaluate how inhibitory plasticity interacts with plastic excitation, we deactivated the plasticity for specific synapses for three model variations. First, we deactivated the plasticity only in the inhibitory feedback connections (*fix fb inh*). Second, the plasticity was deactivated in both excitatory connections the inhibitory population (*fix ff inh*). We further deactivated the plasticity in the connections from the excitatory to the inhibitory population and for the lateral inhibition. Additionally, we trained one model variation where all connections were plastic, to validate that the learning is successful with pre-trained, shuffled weight matrices. To ensure that the same average amount of excitatory or inhibitory current is conveyed by the fixed synapses, we used shuffled weight matrices from previous simulations of the *EI*2/1 model for the respective synapses. No parameter changes were needed. To test the stability and reproducibility, we performed five runs of each variation.

### Analysis methods

#### Receptive field mapping

Over the course of learning, the excitatory input weights from LGN to V1 develop based on the pre- and postsynaptic activity. It is therefore possible to obtain a good approximation of the neurons’ receptive fields (RFs) by taking the weight matrix and reverting the ON-OFF mapping. To do this, we subtract the OFF-synapses from the ON-synapses and receive the receptive field. This is possible as either the ON- or the OFF-synapses can be activated by the input, so that the weights will also follow this distribution.

In addition to the visualization based on weight matrices, the receptive fields can also be revealed by probing the neurons with random stimuli. This approach has been successfully used in physiological research, in form of the spike triggered average (STA) [108, 109, 110]. In this method, a neuron’s receptive field is defined as the average of white noise stimuli, weighted by the stimulus-triggered neuronal activity. We applied this method on the learned neural network. We presented noise patches drawn from a normal distribution with *μ* = 15, *σ* = 20 as input image to the network, and converted these to Poisson spike trains (cf. Sec. **Network input**). Negative pixel values were set to zero, and the presentation time per patch was 125*ms*. For each neuron, we recorded the number of spikes per stimulus and calculated the average across all stimuli, weighted by the number of postsynaptic spikes (Eq 12).

$$\begin{array}{c} STA=\frac{1}{N}\sum_{n=1}^{N}s\left(t_{n}\right) \end{array}$$ (12)

Here, *s*(*t*_*n*_) is the input stimulus at time point *t*_*n*_, when the *n*th spike has occurred, and *N* is the total number of postsynaptic spikes. Accordingly, stimuli evoking more spikes are higher weighted than stimuli evoking few or no spikes.

As we observed a high similarity between each neuron’s STA and its ON-OFF receptive field, we concluded that the overall receptive field shape was not significantly influenced by inhibition. Thus, for simplicity, the feed-forward weight vectors can be used for further evaluations.

#### Receptive field similarity

As mentioned above, the feed-forward weight vector approximates the receptive field of a neuron. To measure the similarity between two receptive fields, we calculate the cosine between their feed-forward weight vectors (Eq 13).

$$\begin{array}{c} \text{cos}\left(\phi_{i,j}\right)=\frac{W_{i}\cdot W_{j}}{|W_{i}\left|\right|W_{j}|} \end{array}$$ (13)

A value near + 1 indicates high similarity, values around zero describe orthogonal weight vectors, and values near −1 indicates inverted weight vectors (i.e., maximally overlapping RFs with opposite directional preference).

#### Tuning curves and orientation selectivity

The orientation selectivity is a well-studied characteristic of simple cells in V1 of mammals [17, 111, 112] and thus, also a topic of interest for models of the visual cortex [e.g., 113], [114], [115]. One possibility to quantify the orientation selectivity of a neuron is to measure its tuning curve [116]. For simple cells in the primary visual cortex, the orientation tuning curve describes the magnitude of responses evoked by a stimulus presented at different angles. In many biological studies, the tuning curves have been measured based on two-dimensional sinusoidal gratings [36, 75, 76, 116]. Therefore, we measured the responses to sinusoidal grating stimuli, rotated in steps of 8°, with different spatial phases from 0*rad* to *πrad*, a different spatial frequencies from 0.05 up to 0.15*cycles*/*pixel*, centred to the input space and with a presentation time of 125*ms*.

Because of Poisson activity in the input layer, neuronal activity shows trial-to-trial fluctuations. Hence, we repeated every presentation 50 times, and calculated the mean across all 50 repetitions (or 6.25*s* presentation time). In contrast to the natural scene input used for training, the maximum input firing rate was set to 85.7*Hz*. This was suitable to obtain sufficiently high activity levels.

To estimate tuning curve sharpness, we calculated the orientation bandwidth (OBW) for every neuron. The OBW is defined as the half-width of the tuning curve, at an activity level of $\frac{1}{\sqrt{2}}$ (approx. 70.7%) of the maximum [116]. Higher OBW values correspond to a broader tuning curve, and vice versa. Other definitions use the height at half-maximum, which does not change the overall result of this evaluation.

#### Orientation diversity

To quantify the diversity of receptive field orientations, we calculated a histogram over the measured preferred orientations to measure the distribution and the incidence of a specific orientation (*P*(*o*) where *o* is the index to a specific orientation) Then, we calculated the Kullback-Leibler divergence (Eq 14) between this distribution and an idealized uniform distribution of orientations (*Q*(*o*)).

$$\begin{array}{c} D_{KL}\left(P\|Q\right)=\sum_{o=1}P\left(o\right)log(\frac{P(o)}{Q(o)}) \end{array}$$ (14)

$$\begin{array}{c} ODI={\text{exp}}^{-D_{KL}\left(P\|Q\right)} \end{array}$$ (15)

To calculate the orientation diversity index (*ODI*), we used the exponential function on the calculated Kullback-Leibler divergence. A value closer to one indicates a more uniform distribution of the measured orientations and thus a higher orientation diversity, whereas a value closer to zero indicates a less uniform distribution and thus a lower orientation diversity.

#### Neuronal gain curves

A neuron’s gain function describes how neuronal activity is scaled by variations in the magnitude of excitatory inputs [5, 75]. While an integrate-and-fire neuron receiving only excitatory inputs has a relatively static gain function (also called transfer function), controlled by the parameters of the neuron model, additional inhibitory inputs can modulate the effective input-to-output relationship. To characterize these inhibitory influences on gain curves, we recorded the excitatory synaptic currents and spiking activity evoked by sinus gratings (see Sec. **Tuning curves and orientation selectivity**), which we rotated from the orthogonal towards the preferred orientation of each neuron. Further, we changed the contrast of the input, by changing the pixels relative to the maximum input firing from 14.25*Hz* up to 100*Hz*. As before, we presented each stimulus orientation for 125*ms*, repeated 50 times (6.25*s*), and determined gain curves based on the average spike count across these 50 repetitions. We measured the spike count for each input degree and contrast strength and sorted the neuronal activity to the corresponding excitatory input, in ascending order.

#### Measurement of E to I ratio

To determine the ratio between excitatory and inhibitory input current, we measure both incoming currents for the excitatory population for 1.000 randomly chosen natural scenes. Every scene was presented for 125*ms* and was repeatedly shown for 100 times. We averaged the incoming currents over the input stimuli repetitions and sorted for each neuron and stimuli the excitatory input currents ascending with the related inhibitory currents. For better visualization, the currents are summarized into bins.

#### Sparseness

The sparseness value expresses the specificity of population codes and single neurons, both in experimental studies [40, 81, 82, 83, 117] and in model simulations [8, 13, 35]. It quantifies either the fraction of neurons which respond to a single stimulus, called population sparseness, or the number of stimuli to which a single neuron responds, called lifetime sparseness [83]. In the past, many different sparseness measurements are established [81, 118]. To measure the specificity of our network activity, we calculated the population sparseness after Vinje and Gallant [82] (see Eq 16).

$$\begin{array}{c} S=\frac{1-\frac{{\left(\sum r_{i}/n\right)}^{2}}{\sum (r_{i}^{2}/n)}}{1-(1/n)} \end{array}$$ (16)

where *r*_*i*_ is the activity of the *i*th neuron to a specific input and *n* the number of neurons in the neuron population.

By construction, sparseness values are bound between zero and one. If the neuron population has dense activity, i.e., most neurons are active to an input stimulus, the sparseness level approaches zero. By contrast, few active neurons of the population lead to a sparseness value close to one. As input, we used 30.000 natural scene patches, and determined sparseness values based on the firing rates of each neuron on each input patch.

#### Image reconstruction error

The network’s coding performance following training can be measured by the difference between input images and their reconstruction from network activity. This method gives direct insight on how well visual input is represented by the network as a whole. This aspect was often not considered in previous biologically motivated circuit models of the primary visual cortex. We used the root mean-square error between one image of the natural scenes dataset from [6] and the reconstructed one [cf. 13, 119] (Eq 17), termed image reconstruction error (IRE):

$$\begin{array}{c} IRE=\sqrt{\frac{\sum_{k}{\left(I_{o}-I_{r}\right)}^{2}}{N}} \end{array}$$ (17)

where *N* denotes the number of image pixels. To obtain the reconstructed image *I*_*r*_, we subdivided the full image into patches of size 12 × 12, in an overlapping fashion (in increments of 3 pixels). We showed each patch 50 times for 125*ms* each, and recorded neuronal activities. We weighted the activity of each neuron by its feed-forward weights to obtain a linear reconstruction of each image patch, which we combined to reconstruct the full image. This approach is equivalent to calculating the IRE for individual patches, and calculating the root mean-square of these individual IRE values. To ensure that pixel values of the reconstructed image were in the same range as the original image, we normalized the reconstructed as well as the original image to zero mean and unit variance [13, 119].

#### Mutual information

We measure the metabolic efficiency via the numbers of spikes which are necessary to represent a specific input stimuli and the amount of information transmitted via a spike. An information-theoretic approach to estimate this coding efficiency of the network is based on the mutual information between stimulus identity and neuronal activity [2, 120]. This measure allows to calculate the average information transmission per spike [117, 121]. To quantify information transmission, we calculated the mutual information, *I*(*s*, *r*), between the stimulus identity and neuronal responses for each neuron, following Vinje and Gallant [117]:

$$\begin{array}{c} I(s,r)=H(r)-H(r|s) \end{array}$$ (18)

In Eq 18, *I*(*s*, *r*) is the mutual information carried between stimulus and response for a time bin of 125*ms* length, the duration of a single stimulus. For that purpose, we calculate the total response entropy, *H*(*r*), and the conditional response entropy, also called stimulus-specific noise entropy, *H*(*r*|*s*).

$$\begin{array}{c} H\left(r\right)=-\sum_{j=0}^{\infty }p_{j}log_{2}\left(p_{j}\right) \end{array}$$ (19)

$$\begin{array}{c} H\left(r|s=k\right)=-\sum_{j=0}^{\infty }p_{j}^{k}log_{2}\left(p_{j}^{k}\right) \end{array}$$ (20)

The total response entropy is given by Eq 19. The variable *p*_*j*_ is the number of time bins containing exactly *j* spikes, divided by the total number of time bins, or stimuli. It follows from Eq 19 that the total response entropy is maximal if all spike counts occur with equal probability (and, if they do, the number of possible spike counts increases the entropy). The noise entropy for a specific stimulus (see Eq 20) describes the variability of the neuronal responses across repetitions of a single stimulus *k*. Every stimulus was repeated 100 times. Similar to the total response entropy, *j* is the number of spikes which occurred in response to a stimulus *k*. Here, $p_{j}^{k}$ is the number of repetitions of stimulus *k* to which exactly *j* spikes are emitted, divided by the overall number of repetitions of that stimulus. To calculate the overall noise entropy of a neuron *H*(*r*|*s*), we averaged the noise entropy across all stimuli. Information per spike was computed by dividing *I*(*s*, *r*) by the mean number of spikes per stimuli, or time bins.

#### Discriminability

To evaluate how well the network responses allow to distinguish between any two input patches, in the presence of trial-to-trial (how much is the variance in the firing rate of a neuron to specific input [122]) fluctuations induced by Poisson input, we calculated the discriminability index, *d*′ [2, 120]. The *d*′ index measures the separation of two random distributions, and is closely related to the performance of a linear classifier assuming independent neuronal responses. Based on a random set of 500 natural scene patches, we calculated the *d*′ by pairing the response on every patch to all other patches. For each pair of stimuli, *s*_1_ and *s*_2_, we presented each stimulus with *N* = 100 repetitions, and recorded the network responses of all *n* = 144 excitatory neurons for each repetition, obtaining the *n*-dimensional response vectors $s_{1}^{\left(i\right)}$ and $s_{2}^{\left(i\right)}$, *i* = 1, …, *N*. We first calculated the mean activity of each cell in response to each stimulus, across the *N* repetitions (denoted by $\bar{s_{1}}$ and $\bar{s_{2}}$). We next projected each individual population response $s_{1}^{\left(i\right)}$ and $s_{2}^{\left(i\right)}$ onto the vector between these means, by taking the dot product between each response and the difference $\bar{s_{1}}-\bar{s_{2}}$:

$$\begin{array}{c} \begin{array}{cc} \alpha_{s_{1}}^{\left(i\right)} & =s_{1}^{\left(i\right)}\cdot \left(\bar{s_{1}}-\bar{s_{2}}\right)\\ \alpha_{s_{2}}^{\left(i\right)} & =s_{2}^{\left(i\right)}\cdot \left(\bar{s_{1}}-\bar{s_{2}}\right)\;\text{for}\;i=1,\ldots ,N \end{array} \end{array}$$ (21)

where $\alpha_{s_{1}}$ and $\alpha_{s_{2}}$ denote the projected responses. Next, we calculated the means and variances of the projected responses $\alpha_{s_{1}}$ and $\alpha_{s_{2}}$, denoted by ($\mu_{s_{1}},\sigma_{s_{1}}^{2}$) and ($\mu_{s_{2}},\sigma_{s_{2}}^{2}$). Finally, we calculate the discriminability $d_{s_{1},s_{2}}^{\prime }$, as the ratio between the separation of the means and the variances of the projected data:

$$\begin{array}{c} d_{s_{1},s_{2}}^{\prime }=\frac{\mu_{s_{1}}-\mu_{s_{2}}}{\sqrt{\frac{1}{2}\left(\sigma_{s_{1}}^{2}+\sigma_{s_{2}}^{2}\right)}} \end{array}$$ (22)

Note that we used the same sequence of patches for all model configurations to calculate the discriminability, and every patch was presented for 125*ms*. Previous research found that the variance of the response of a neuron to input stimuli is proportional to the mean [123]. Further studies demonstrated that inhibition leads to less variance in the responses to one repeatedly shown stimulus [51]. The discriminability (*d*′) increases if the response variance decreases by the same response mean. Therefore, we can measure differences in the response variance.

## Supporting information

S1 Fig Image reconstruction error (IRE) for different fixed and plastic inhibitory connections. Excitatory synapses learned with the Clopath et al. [20] learning rule. The dark green model (called *base*) is equal with the *EI*2/1 model. The other models are initialized with shuffled weights of a previous successfully learned *EI*2/1 model. In the *plastic inh* model, all inhibitory synapses are plastic, in the *fix ff inh* model is the feed-forward inhibition fixed, in the *fix fb inh* model is the feedback inhibition fixed, and in the in the *non plastic* model are all inhibitory connections fixed.
(TIF)

S2 Fig Development of receptive fields. Input weights of five randomly chosen excitatory cells. Bright values show input from the ON-LGN population and dark values from the OFF-LGN population. ON and the OFF weights are subtracted from each other to show the receptive fields. **A** Emergence of stable receptive fields. **B** Examples of unstable receptive fields, i.e when the differences between ON and OFF weights are zero (gray values) due to the increase of both components to the maximum value.
(TIF)

S3 Fig Dynamics of weights during the first 200, 000 stimulus presentations. First column shows stable weight learning. Second column shows the unlimited growth of the weights against the maximum weight value. **A** and **B** mean feed-forward excitatory weights from the LGN population to one excitatory neuron. **C** and **D** inhibitory feedback weights from the inhibitory population to one excitatory neuron. **E** and **F** mean input currents of the excitatory population.
(TIF)

S4 Fig Histogram of feed-forwards weights. Histogram of feed-forwards weights from the LGN to the excitatory population **A**, and of the feed-forwards weights from the LGN to the inhibitory population **B** from the *EI*2/1 model.
(TIF)

S5 Fig Receptive fields and orientation distribution from the *EI*3/1 model. **A** Receptive fields of randomly selected 64 excitatory neurons of the *EI*3/1 model. **B** Distribution of receptive field orientation of all excitatory neurons of 20 model runs (*EI*3/1 model). **C** Receptive fields of all 36 inhibitory neurons of the *EI*3/1 model. **D** Distribution of receptive field orientation of all inhibitory neurons of 20 model runs (*EI*3/1 model).
(TIF)

S6 Fig Average discriminability. Average discriminability (*d*′) based on the responses to 500 randomly chosen natural scene patches. Discriminability benefits from tuning diversity of receptive fields and from feedback inhibition.
(TIF)

S7 Fig Orientation tuning as a function of input contrast, *EI*2/1 model. Mean spike count (upper left), average membrane potential (upper middle), standard deviation (upper right), mean excitatory input (lower left), mean inhibitory input (lower middle), and difference between excitation and inhibition (lower right).
(TIF)

S8 Fig Orientation tuning as a function of input contrast, *EI*3/1 model. Mean spike count (upper left), average membrane potential (upper middle), standard deviation (upper right), mean excitatory input (lower left), mean inhibitory input (lower middle), and difference between excitation and inhibition (lower right).
(TIF)

S9 Fig Normalized tuning curves. Tuning curves are normalized with the maximum spike count on high contrast. **A** for *EI*2/1 model, **B** the *EI*3/1 model, Mean and standard deviation calculated across the excitatory population.
(TIF)

S10 Fig Mean orientation bandwidth of the excitatory population for different contrast levels. Green: *EI*2/1 model. Orange: Deactivated inhibition, blue: Randomly shuffled feed-forward and feedback inhibition.
(TIF)

S11 Fig Statistic over stable and unstable receptive fields. **A** Percent of runs in which stable receptive fields or unstable (eliminated) receptive fields emerged during learning for different values of *η* (learning rate) of the Vogels et al. [21] learning rule. Other parameters are taken from the *EI*2/2 model configuration. **B** Percent of runs, where stable receptive fields or unstable (eliminated) receptive fields emerged during learning for different *ρ* (postsynaptic target rate) of the Vogels et al. [21] learning rule. Other parameters are taken from the *EI*2/2 model configuration. Please note, that *ρ* = 0.7 corresponds to the *EI*3/1 model.
(TIF)

S12 Fig Different sized excitatory and inhibitory populations. **A** Image reconstruction error (IRE) as a function of orientation diversity. **B** Orientation Bandwidth (OBW) for different contrast levels. Data from the *EI*2/1 model (green), with twice the number of neurons (red) and with ten times the number of neurons (blue). Note: The number of inhibitory neurons is chosen to fit the 4 : 1 excitation to inhibition ratio.
(TIF)

S13 Fig Weak lateral excitation. Recurrent weights are chosen randomly from a normal distribution with *μ* = 0 and different values of *σ* to control the maximum weight. Negative weight values are set to zero. Blue indicates a maximum weight value of 0.075, olive green indicates a maximum weight value of 0.05, red indicates a maximum weight value of 0.025 and orange indicates a maximum weight values of 0.01. Dark green indicates the *EI*2/1, which is presented for comparison. **A** Percentage of simulations where learning was successful (stable receptive fields emerged) and not successful (all weights in the network run against the maximum weight values). **B** Recurrent excitatory input current as a function of the excitatory current over feed-forward synapses. **C** IRE as a function of the ODIe. **D** OBW for sinusoidal gratings on different levels of contrast.
(TIF)

S14 Fig IRE in a network, trained with the Pfister & Gerstner (2006) learning rule. Image reconstruction error (IRE) as a function of orientation diversity. Excitatory synapses learned with the Pfister & Gerstner (2006) [56] STDP learning rule. Points mark the mean values and the whiskers the standard deviation across 10 model runs.
(TIF)

S15 Fig Image reconstruction error (IRE) as a function of the strength of withe noise. Noise is generated via a normal distribution and added to the natural scene input. The strength is in relation to the maximum pixel value of the original input. Values showing the average IRE of 20 runs for each model configuration, the shaded area represents the standard deviation.
(TIF)

## References

[1] Froudarakis E, Berens P, Ecker AS, Cotton RJ, Sinz FH, Yatsenko D, et al. Population code in mouse V1 facilitates readout of natural scenes through increased sparseness. Nat Neurosci. 2014;17(6):851–7. doi: 10.1038/nn.3707 24747577 PMC4106281

[2] Dadarlat MC, Stryker MP. Locomotion Enhances Neural Encoding of Visual Stimuli in Mouse V1. J Neurosci. 2017;37(14):3764–3775. doi: 10.1523/JNEUROSCI.2728-16.2017 28264980 PMC5394894

[3] Goris RLT, Simoncelli EP, Movshon JA. Origin and Function of Tuning Diversity in Macaque Visual Cortex. Neuron. 2015;88(4):819–831. doi: 10.1016/j.neuron.2015.10.009 26549331 PMC4786576

[4] Carvalho TP, Buonomano DV. Differential Effects of Excitatory and Inhibitory Plasticity on Synaptically Driven Neuronal Input-Output Functions. Neuron. 2009;61(5):774–785. doi: 10.1016/j.neuron.2009.01.013 19285473 PMC2676350

[5] Isaacson JS, Scanziani M. How inhibition shapes cortical activity. Neuron. 2011;72(2):231–243. doi: 10.1016/j.neuron.2011.09.027 22017986 PMC3236361

[6] Olshausen BA, Field DJ. Emergence of simple-cell receptive field properties by learning a sparse code for natural images. Nature. 1996;381(6583):607–609. doi: 10.1038/381607a0 8637596

[7] Bell AJ, Sejnowski TJ. The “independent components” of natural scenes are edge filters. Vision Res. 1997;37(23):3327–3338. doi: 10.1016/S0042-6989(97)00121-1 9425547 PMC2882863

[8] Zylberberg J, Murphy JT, DeWeese MR. A Sparse Coding Model with Synaptically Local Plasticity and Spiking Neurons Can Account for the Diverse Shapes of V1 Simple Cell Receptive Fields. PLoS Comput Biol. 2011;7(10):e1002250. doi: 10.1371/journal.pcbi.1002250 22046123 PMC3203062

[9] Khan AG, Poort J, Chadwick A, Blot A, Sahani M, Mrsic-Flogel TD, et al. Distinct learning-induced changes in stimulus selectivity and interactions of GABAergic interneuron classes in visual cortex. Nat Neurosci. 2018;21(6):851–859. doi: 10.1038/s41593-018-0143-z 29786081 PMC6390950

[10] Wang L, Maffei A. Inhibitory Plasticity Dictates the Sign of Plasticity at Excitatory Synapses. J Neurosci. 2014;34(4):1083–1093. doi: 10.1523/JNEUROSCI.4711-13.2014 24453301 PMC3898280

[11] Mongillo G, Loewenstein Y. Inhibitory connectivity defines the realm of excitatory plasticity. Nat Neurosci. 2018;21(January):1463–1470. doi: 10.1038/s41593-018-0226-x 30224809

[12] Savin C, Joshi P, Triesch J. Independent Component Analysis in Spiking Neurons. PLoS Comput Biol. 2010;6(4):e1000757. doi: 10.1371/journal.pcbi.1000757 20421937 PMC2858697

[13] King PD, Zylberberg J, DeWeese MR. Inhibitory Interneurons Decorrelate Excitatory Cells to Drive Sparse Code formation in a Spiking Model of V1. J Neurosci, 5475. 2013;33(13):5475–5485. doi: 10.1523/JNEUROSCI.4188-12.2013 PMC6705060 23536063

[14] Sadeh S, Clopath C, Rotter S. Emergence of Functional Specificity in Balanced Networks with Synaptic Plasticity. PLoS Comput Biol. 2015;11(6):1–27. doi: 10.1371/journal.pcbi.1004307 26090844 PMC4474917

[15] Miconi T, McKinstry JL, Edelman GM. Spontaneous emergence of fast attractor dynamics in a model of developing primary visual cortex. Nat Commun. 2016;7:13208. doi: 10.1038/ncomms13208 27796298 PMC5095518

[16] Markram H, Toledo-Rodriguez M, Wang Y, Gupta A, Silberberg G, Wu C. Interneurons of the neocortical inhibitory system. Nat Rev Neurosci. 2004;5(10):793–807. doi: 10.1038/nrn1519 15378039

[17] Priebe NJ, Ferster D. Inhibition, Spike Threshold, and Stimulus Selectivity in Primary Visual Cortex. Neuron. 2008;57(4):482–497. doi: 10.1016/j.neuron.2008.02.005 18304479

[18] Beaulieu C, Kisvarday Z, Somogyi P, Cynader M, Cowey A. Quantitative Distribution of GABA-immunopositive and-immunonegative Neurons and Synapses in the Monkey Striate Cortex (Area 17). Cereb Cortex. 1992;2(4):295–309. doi: 10.1093/cercor/2.4.295 1330121

[19] Potjans TC, Diesmann M. The Cell-Type Specific Cortical Microcircuit: Relating Structure and Activity in a Full-Scale Spiking Network Model. Cereb Cortex. 2014;(March):785–806. doi: 10.1093/cercor/bhs358 23203991 PMC3920768

[20] Clopath C, Büsing L, Vasilaki E, Gerstner W. Connectivity reflects coding: a model of voltage-based STDP with homeostasis. Nat Neurosci. 2010;13(3):344–352. doi: 10.1038/nn.2479 20098420

[21] Vogels TP, Sprekeler H, Zenke F, Clopath C, Gerstner W. Inhibitory Plasticity Balances Excitation and Inhibition in Sensory Pathways and Memory Networks. Science. 2011;334(6062):1569–1573. doi: 10.1126/science.1211095 22075724

[22] Jones JP, Palmer La. An Evaluation of the Two-Dimensional Gabor Filter Model of Simple Receptive Fields in Cat Striate Cortex. J Neurophysiol. 1987;58(6):1233–1258. citeulike-article-id:762473. doi: 10.1152/jn.1987.58.6.1187 3437332

[23] Ringach DL. Spatial Structure and Symmetry of Simple-Cell Reveptive Fields in Macaque Primary Visual Cortex. J Neurophysiol. 2002;88(1):455–463. doi: 10.1152/jn.2002.88.1.455 12091567

[24] Spratling MW. Unsupervised Learning of Generative and Discriminative Weights Encoding Elementary Image Components in a Predictive Coding Model of Cortical Function. Neural Comput. 2012;24(1):60–103. doi: 10.1162/NECO_a_00222 22023197

[25] Rose D, Blakemore C. An analysis of orientation selectivity in the cat’s visual cortex. Exp Brain Res. 1974;20(1):1–17. doi: 10.1007/BF00239014 4844166

[26] Chino YM, Shansky MS, Pizzi WJ. Receptive field properties of simple and complex striate neurons in Siamese cats. J Comp Neurol. 1980;190(1):63–86. doi: 10.1002/cne.901900106 7381055

[27] Berman NE, Wilkes ME, Payne BR. Organization of orientation and direction selectivity in areas 17 and 18 of cat cerebral cortex. J Neurophysiol. 1987;58(4):676–699. doi: 10.1152/jn.1987.58.4.676 3316523

[28] Wilson DE, Smith GB, Jacob AL, Walker T, Dimidschstein J, Fishell G, et al. GABAergic Neurons in Ferret Visual Cortex Participate in Functionally Specific Networks. Neuron. 2017;93(5):1058–1065.e4. doi: 10.1016/j.neuron.2017.02.035 28279352 PMC5477844

[29] Kerlin AM, Andermann ML, Berezovskii VK, Reid RC. Broadly Tuned Response Properties of Diverse Inhibitory Neuron Subtypes in Mouse Visual Cortex. Neuron. 2010;67(5):858–871. doi: 10.1016/j.neuron.2010.08.002 20826316 PMC3327881

[30] Hofer SB, Ko H, Pichler B, Vogelstein J, Ros H, Zeng H, et al. Differential connectivity and response dynamics of excitatory and inhibitory neurons in visual cortex. Nat Neurosci. 2011;14:1045 EP –. doi: 10.1038/nn.2876 21765421 PMC6370002

[31] Bock DD, Lee WCA, Kerlin AM, Andermann ML, Hood G, Wetzel AW, et al. Network anatomy and in vivo physiology of visual cortical neurons. Nature. 2011;471:177 EP –. doi: 10.1038/nature09802 21390124 PMC3095821

[32] Liu Bh, Li Yt, Ma Wp, Pan Cj, Zhang LI, Tao HW. Broad inhibition sharpens orientation selectivity by expanding input dynamic range in mouse simple cells. Neuron. 2011;71(3):542–554. doi: 10.1016/j.neuron.2011.06.017 21835349 PMC3154747

[33] Runyan CA, Schummers J, Van Wart A, Kuhlman SJ, Wilson NR, Huang ZJ, et al. Response Features of Parvalbumin-Expressing Interneurons Suggest Precise Roles for Subtypes of Inhibition in Visual Cortex. Neuron. 2010;67(5):847–857. doi: 10.1016/j.neuron.2010.08.006 20826315 PMC2948796

[34] Cossell L, Iacaruso MF, Muir DR, Houlton R, Sader EN, Ko H, et al. Functional organization of excitatory synaptic strength in primary visual cortex. Nature. 2015;000(00):1–5. doi: 10.1038/nature14182 25652823 PMC4843963

[35] Wiltschut J, Hamker FH. Efficient coding correlates with spatial frequency tuning in a model of V1 receptive field organization. Vis Neurosci. 2009;26(1):21–34. doi: 10.1017/S0952523809090051 19203427

[36] Smith MA, Kohn A. Spatial and temporal scales of neuronal correlation in primary visual cortex. J Neurosci. 2008;28(48):12591–603. doi: 10.1523/JNEUROSCI.2929-08.2008 19036953 PMC2656500

[37] Kayser C, Salazar RF, König P. Responses to Natural Scenes in Cat V1. J Neurophysiol. 2003;90(3):1910–1920. doi: 10.1152/jn.00195.2003 12750423

[38] Denman DJ, Contreras D. The Structure of Pairwise Correlation in Mouse Primary Visual Cortex Reveals Functional Organization in the Absence of an Orientation Map. Cereb Cortex. 2013;24(10):2707–2720. doi: 10.1093/cercor/bht128 23689635 PMC4153809

[39] Martin KAC, Schröder S. Functional Heterogeneity in Neighboring Neurons of Cat Primary Visual Cortex in Response to Both Artificial and Natural Stimuli. J Neurosci. 2013;33(17):7325–7344. doi: 10.1523/JNEUROSCI.4071-12.2013 23616540 PMC6619576

[40] Weliky M, Fiser J, Hunt RH, Wagner DN. Coding of natural scenes in primary visual cortex. Neuron. 2003;37(4):703–18. doi: 10.1016/S0896-6273(03)00022-9 12597866

[41] Averbeck BB, Latham PE, Pouget A. Neural correlations, population coding and computation. Nat Rev Neurosci. 2006;7(May). 16760916 10.1038/nrn1888

[42] Sippy T, Yuste R. Decorrelating Action of Inhibition in Neocortical Networks. J Neurosci. 2013;33(23):9813–9830. doi: 10.1523/JNEUROSCI.4579-12.2013 23739978 PMC3715137

[43] Stringer C, Pachitariu M, Steinmetz NA, Okun M, Bartho P, Harris KD, et al. Inhibitory control of correlated intrinsic variability in cortical networks. eLife. 2016;5:e19695. doi: 10.7554/eLife.19695 27926356 PMC5142814

[44] Ben-Yishai R, Bar-Or RL, Sompolinsky H. Theory of orientation tuning in visual cortex. Proceedings of the National Academy of Sciences. 1995;92(9):3844–3848. doi: 10.1073/pnas.92.9.3844 7731993 PMC42058

[45] Troyer TW, Krukowski AE, Priebe NJ, Miller KD. Contrast-Invariant Orientation Tuning in Cat Visual Cortex: Thalamocortical Input Tuning and Correlation-Based Intracortical Connectivity. J Neurosci. 1998;18(15):5908–5927. doi: 10.1523/JNEUROSCI.18-15-05908.1998 9671678 PMC6793055

[46] Ferster D, Miller KD. Neural Mechanisms of Orientation Selectivity in the Visual Cortex. Annu Rev Neurosci. 2000;23(1):441–471. doi: 10.1146/annurev.neuro.23.1.441 10845071

[47] Skottun BC, Bradley A, Sclar G, Ohzawa I, Freeman RD. The effects of contrast on visual orientation and spatial frequency discrimination: a comparison of single cells and behavior. J Neurophysiol. 1987;57(3):773–786. doi: 10.1152/jn.1987.57.3.773 3559701

[48] Finn IM, Priebe NJ, Ferster D. The Emergence of Contrast-Invariant Orientation Tuning in Simple Cells of Cat Visual Cortex. Neuron. 2007;54(1):137–152. doi: 10.1016/j.neuron.2007.02.029 17408583 PMC1993919

[49] Alitto HJ, Usrey WM. Influence of Contrast on Orientation and Temporal Frequency Tuning in Ferret Primary Visual Cortex. J Neurophysiol. 2004;91(6):2797–2808. doi: 10.1152/jn.00943.2003 14762157

[50] Spanne A, Jörntell H. Questioning the role of sparse coding in the brain. Trends Neurosci. 2015;38(7):417–427. doi: 10.1016/j.tins.2015.05.005 26093844

[51] Haider B, Krause MR, Duque A, Yu Y, Touryan J, Mazer JA, et al. Synaptic and Network Mechanisms of Sparse and Reliable Visual Cortical Activity during Nonclassical Receptive Field Stimulation. Neuron. 2010;65(1):107–121. doi: 10.1016/j.neuron.2009.12.005 20152117 PMC3110675

[52] Zhu W, Shelley M, Shapley R. A neuronal network model of primary visual cortex explains spatial frequency selectivity. J Comput Neurosci. 2009;26(2):271–287. doi: 10.1007/s10827-008-0110-x 18668360

[53] Kremkow J, Perrinet LU, Monier C, Alonso JM, Aertsen A, Frégnac Y, et al. Push-Pull Receptive Field Organization and Synaptic Depression: Mechanisms for Reliably Encoding Naturalistic Stimuli in V1. Front Neural Circuits. 2016;10(May):37. doi: 10.3389/fncir.2016.00037 27242445 PMC4862982

[54] Graham DJ, Field DJ. Natural Images: Coding Efficiency. Encyclopedia of Neuroscience. 2010;6:19–27.

[55] Mitchison G, Barlow HB. Neuronal branching patterns and the economy of cortical wiring. Proceedings of the Royal Society of London Series B: Biological Sciences. 1991;245(1313):151–158. doi: 10.1098/rspb.1991.0102 1682939

[56] Pfister JP, Gerstner W. Triplets of Spikes in a Model of Spike Timing-Dependent Plasticity. J Neurosci. 2006;26(38):9673–9682. doi: 10.1523/JNEUROSCI.1425-06.2006 16988038 PMC6674434

[57] Kermani Kolankeh A, Teichmann M, Hamker FH. Competition improves robustness against loss of information. Front Comput Neurosci. 2015;9(March):35. doi: 10.3389/fncom.2015.00035 25859211 PMC4373393

[58] Larisch R, Teichmann M, Hamker FH. A Neural Spiking Approach Compared to Deep Feedforward Networks on Stepwise Pixel Erasement. In: Kůrková V, Manolopoulos Y, Hammer B, Iliadis L, Maglogiannis I, editors. Artificial Neural Networks and Machine Learning—ICANN 2018. Cham: Springer International Publishing; 2018. p. 253–262.

[59] Griffen T, Maffei A. GABAergic synapses: their plasticity and role in sensory cortex. Front Cell Neurosci. 2014;8:91. doi: 10.3389/fncel.2014.00091 24723851 PMC3972456

[60] Znamenskiy P, Kim MH, Muir DR, Iacaruso MF, Hofer SB, Mrsic-Flogel TD. Functional selectivity and specific connectivity of inhibitory neurons in primary visual cortex. bioRxiv. 2018.

[61] Paille V, Fino E, Du K, Morera-Herreras T, Perez S, Kotaleski JH, et al. GABAergic Circuits Control Spike-Timing-Dependent Plasticity. J Neurosci. 2013;33(22):9353–9363. doi: 10.1523/JNEUROSCI.5796-12.2013 23719804 PMC6618570

[62] Froemke RC, Merzenich MM, Schreiner CE. A synaptic memory trace for cortical receptive field plasticity. Nature. 2007;450(7168):425–429. doi: 10.1038/nature06289 18004384

[63] Kullmann DM, Moreau AW, Bakiri Y, Nicholson E. Plasticity of Inhibition. Neuron. 2012;75(6):951–962. doi: 10.1016/j.neuron.2012.07.030 22998865

[64] D’Amour JA, Froemke RCR. Inhibitory and excitatory spike-timing-dependent plasticity in the auditory cortex. Neuron. 2015;86(2):514–528. doi: 10.1016/j.neuron.2015.03.014 25843405 PMC4409545

[65] Litwin-Kumar A, Doiron B. Formation and maintenance of neuronal assemblies through synaptic plasticity. Nat Commun. 2014;5(May):1–12. 25395015 10.1038/ncomms6319

[66] Sprekeler H. Functional consequences of inhibitory plasticity: homeostasis, the excitation-inhibition balance and beyond. Curr Opin Neurobiol. 2017;43:198–203. doi: 10.1016/j.conb.2017.03.014 28500933

[67] Srinivasa N, Jiang Q. Stable learning of functional maps in self-organizing spiking neural networks with continuous synaptic plasticity. Front Comput Neurosci. 2013;7:10. doi: 10.3389/fncom.2013.00010 23450808 PMC3583036

[68] Hennequin G, Agnes EJ, Vogels TP. Inhibitory Plasticity: Balance, Control, and Codependence. Annu Rev Neurosci. 2017;40(1):557–579. doi: 10.1146/annurev-neuro-072116-031005 28598717

[69] Dorrn AL, Yuan K, Barker AJ, Schreiner CE, Froemke RC. Developmental sensory experience balances cortical excitation and inhibition. Nature. 2010;465:932–936. doi: 10.1038/nature09119 20559387 PMC2888507

[70] Froemke RC. Plasticity of Cortical Excitatory-Inhibitory Balance. Annu Rev Neurosci. 2015;38(1):195–219. doi: 10.1146/annurev-neuro-071714-034002 25897875 PMC4652600

[71] Denève S, Machens CK. Efficient codes and balanced networks. Nat Neurosci. 2016;19(3):375–82. doi: 10.1038/nn.4243 26906504

[72] Atallah BV, Bruns W, Carandini M, Scanziani M. Parvalbumin-Expressing Interneurons Linearly Transform Cortical Responses to Visual Stimuli. Neuron. 2012;73(1):159–170. doi: 10.1016/j.neuron.2011.12.013 22243754 PMC3743079

[73] Wilson NR, Runyan CA, Wang FL, Sur M. Division and subtraction by distinct cortical inhibitory networks in vivo. Nature. 2012;488(7411):343–348. doi: 10.1038/nature11347 22878717 PMC3653570

[74] Zhu Y, Qiao W, Liu K, Zhong H, Yao H. Control of response reliability by parvalbumin-expressing interneurons in visual cortex. Nat Commun. 2015;6:1–11. doi: 10.1038/ncomms7802 25869033

[75] Katzner S, Busse L, Carandini M. GABAA Inhibition Controls Response Gain in Visual Cortex. J Neurosci. 2011;31(16):5931–5941. doi: 10.1523/JNEUROSCI.5753-10.2011 21508218 PMC3083851

[76] Anderson JS, Carandini M, Ferster D. Orientation tuning of input conductance, excitation, and inhibition in cat primary visual cortex. J Neurophysiol. 2000;84:909–926. doi: 10.1152/jn.2000.84.2.909 10938316

[77] Adesnik H. Synaptic Mechanisms of Feature Coding in the Visual Cortex of Awake Mice. Neuron. 2017;95(5):1147–1159.e4. doi: 10.1016/j.neuron.2017.08.014 28858618 PMC5580349

[78] Mitchell SJ, Silver RA. Shunting Inhibition Modulates Neuronal Gain during Synaptic Excitation. Neuron. 2003;38(3):433–445. doi: 10.1016/S0896-6273(03)00200-9 12741990

[79] Sadagopan S, Ferster D. Feedforward Origins of Response Variability Underlying Contrast Invariant Orientation Tuning in Cat Visual Cortex. Neuron. 2012;74(5):911–923. doi: 10.1016/j.neuron.2012.05.007 22681694 PMC3591513

[80] Priebe NJ. Mechanisms of Orientation Selectivity in the Primary Visual Cortex. Annu Rev Vis Sci. 2016;2(1):85–107. doi: 10.1146/annurev-vision-111815-114456 28532362

[81] Rolls ET, Tovee MJ. Sparseness of the Neuronal Representation of Stimuli in the Primate Temporal Visual Cortex. J Neurophysiol. 1995;73(2):713–726. doi: 10.1152/jn.1995.73.2.713 7760130

[82] Vinje WE, Gallant JL. Sparse Coding and Decorrelation in Primary Visual Cortex During Natural Vision. Science. 2000;287(5456):1273–1276. doi: 10.1126/science.287.5456.1273 10678835

[83] Tolhurst DJ, Smyth D, Thompson ID. The Sparseness of Neuronal Responses in Ferret Primary Visual Cortex. J Neurosci. 2009;29(8):2355–2370. doi: 10.1523/JNEUROSCI.3869-08.2009 19244512 PMC6666250

[84] Berkes P, White BL, Fiser J. No Evidence for Active Sparsification in the Visual Cortex. In: Proceedings of the 22nd International Conference on Neural Information Processing Systems. NIPS’09. Red Hook, NY, USA: Curran Associates Inc.; 2009. p. 108–116. https://proceedings.neurips.cc/paper/2009/file/2b24d495052a8ce66358eb576b8912c8-Paper.pdf

[85] Barak O, Rigotti M, Fusi S. The Sparseness of Mixed Selectivity Neurons Controls the Generalization-Discrimination Trade-Off. J Neurosci. 2013;33(9):3844–3856. doi: 10.1523/JNEUROSCI.2753-12.2013 23447596 PMC6119179

[86] Harris KD, Mrsic-Flogel TD. Cortical connectivity and sensory coding. Nature. 2013;503:51–58. doi: 10.1038/nature12654 24201278

[87] Ko H, Hofer S, Pichler B, A Buchanan K, Sjöström P, Mrsic-Flogel T. Functional specificity of local connections in neocortical networks. Nature. 2011;473:87–91. doi: 10.1038/nature09880 21478872 PMC3089591

[88] Zenke F, Gerstner W. Hebbian plasticity requires compensatory processes on multiple timescales. Philosophical Transactions of the Royal Society B: Biological Sciences. 2017;372(1715):20160259. doi: 10.1098/rstb.2016.0259 28093557 PMC5247595

[89] Vogels TP, Rajan K, Abbott LF. Neural Network Dynamics. Annu Rev Neurosci. 2005;28(1):357–376. doi: 10.1146/annurev.neuro.28.061604.135637 16022600

[90] Butko NJ, Triesch J. Learning sensory representations with intrinsic plasticity. Neurocomputing. 2007;70(7):1130–1138. doi: 10.1016/j.neucom.2006.11.006

[91] Stevens JLR, Law JS, Antolík J, Bednar JA. Mechanisms for Stable, Robust, and Adaptive Development of Orientation Maps in the Primary Visual Cortex. J Neurosci. 2013;33(40):15747–15766. doi: 10.1523/JNEUROSCI.1037-13.2013 24089483 PMC6618482

[92] Földiák P. Forming sparse representations by local anti-Hebbian learning. Biol Cybern. 1990;64(2):165–170. doi: 10.1007/BF02331346 2291903

[93] Espinosa J S and Stryker M P. Development and Plasticity of the Primary Visual Cortex. Neuron. 2012;75(2):230–249. doi: 10.1016/j.neuron.2012.06.009 22841309 PMC3612584

[94] Toyoizumi T, Miyamoto H, Yazaki-Sugiyama Y, Atapour N, Hensch T, Miller K. A Theory of the Transition to Critical Period Plasticity: Inhibition Selectively Suppresses Spontaneous Activity. Neuron. 2013;80(1):51–63. doi: 10.1016/j.neuron.2013.07.022 24094102 PMC3800182

[95] van Versendaal, Daniëlle and Levelt, Christiaan N. Inhibitory interneurons in visual cortical plasticity. Cell Mol Life Sci. 2016;73:3677–3691. doi: 10.1007/s00018-016-2264-4 27193323 PMC5002041

[96] Issa NP, Trachtenberg JT, Chapman B, Zahs KR, Stryker MP. The Critical Period for Ocular Dominance Plasticity in the Ferret’s Visual Cortex. J Neurosci. 1999;19(16):6965–6978. doi: 10.1523/JNEUROSCI.19-16-06965.1999 10436053 PMC2413141

[97] Gandhi SP, Yanagawa Y, Stryker MP. Delayed plasticity of inhibitory neurons in developing visual cortex. Proceedings of the National Academy of Sciences. 2008;105(43):16797–16802. doi: 10.1073/pnas.0806159105 18940923 PMC2575499

[98] Kuhlman SJ, Olivas ND, Tring E, Ikrar T, Xu X, Trachtenberg JT. A disinhibitory microcircuit initiates critical-period plasticity in the visual cortex. Nature. 2013;501(7468):543–546. doi: 10.1038/nature12485 23975100 PMC3962838

[99] Gaudry KS, Reinagel P. Benefits of contrast normalization demonstrated in neurons and model cells. J Neurosci 2007;27(30):8071–8079. doi: 10.1523/JNEUROSCI.1093-07.2007 17652598 PMC6672724

[100] Vitay J, Dinkelbach H, Hamker F. ANNarchy: a code generation approach to neural simulations on parallel hardware. Front Neuroinform. 2015;9:19. doi: 10.3389/fninf.2015.00019 26283957 PMC4521356

[101] Larisch R. [Re] Connectivity reflects coding a model of voltage-based STDP with homeostasis. ReScience C. 2019;5(3). 10.1038/nn.2479 20098420

[102] Andrews TJ, Halpern SD, Purves D. Correlated Size Variations in Human Visual Cortex, Lateral Geniculate Nucleus, and Optic Tract. J Neurosci. 1997;17(8):2859–2868. doi: 10.1523/JNEUROSCI.17-08-02859.1997 9092607 PMC6573115

[103] Ko H, Cossell L, Baragli C, Antolik J, Clopath C, Hofer SB, et al. The emergence of functional microcircuits in visual cortex. Nature. 2013;496(7443):96–100. doi: 10.1038/nature12015 23552948 PMC4843961

[104] Lee WCA, Bonin V, Reed M, Graham BJ, Hood G, Glattfelder K, et al. Anatomy and function of an excitatory network in the visual cortex. Nature. 2016;532(7599):370–374. doi: 10.1038/nature17192 27018655 PMC4844839

[105] Olshausen BA, Field DJ. Sparse coding with an overcomplete basis set: A strategy employed by V1? Vision Res. 1997;37(23):3311–3325. 9425546 10.1016/s0042-6989(97)00169-7

[106] Bienenstock EL, Cooper LN, Munro PW. Theory for the development of neuron selectivity: orientation specificity and binocular interaction in visual cortex. J Neurosci. 1982;2(1):32–48. doi: 10.1523/JNEUROSCI.02-01-00032.1982 7054394 PMC6564292

[107] Caporale N, Dan Y. Spike Timing–Dependent Plasticity: A Hebbian Learning Rule. Annu Rev Neurosci. 2008;31:25–46. doi: 10.1146/annurev.neuro.31.060407.125639 18275283

[108] Ringach DL, Shapley R. Reverse correlation in neurophysiology. Cogn Sci. 2004;28(2):147–166. doi: 10.1207/s15516709cog2802_2

[109] Schwartz O, Pillow JW, Rust NC, Simoncelli EP. Spike-triggered neural characterization. J Vis. 6,. 2006;6(4):484–507. 16889482 10.1167/6.4.13

[110] Pillow JW, Simoncelli EP. Dimensionality reduction in neural models: an information-theoretic generalization of spike-triggered average and covariance analysis. J Vis. 2006;6(4):414–428. doi: 10.1167/6.4.9 16889478

[111] Gilbert CD, Wiesel TN. The influence of contextual stimuli on the orientation selectivity of cells in primary visual cortex of the cat. Vision Res. 1990;30(11):1689–701. doi: 10.1016/0042-6989(90)90153-C 2288084

[112] Niell CM, Stryker MP. Highly Selective Receptive Fields in Mouse Visual Cortex. J Neurosci. 2008;28(30):7520–7536. doi: 10.1523/JNEUROSCI.0623-08.2008 18650330 PMC3040721

[113] Sadeh S, Cardanobile S, Rotter S. Mean-field analysis of orientation selectivity in inhibition-dominated networks of spiking neurons. SpringerPlus. 2014;3(1):148. doi: 10.1186/2193-1801-3-148 24790806 PMC4003001

[114] Zhu W, Xing D, Shelley M, Shapley R. Correlation between spatial frequency and orientation selectivity in V1 cortex: Implications of a network model. Vision Res. 2010;50(22):2261–2273. doi: 10.1016/j.visres.2010.01.007 20079759 PMC2904434

[115] Tao L, Shelley M, McLaughlin D, Shapley R. An egalitarian network model for the emergence of simple and complex cells in visual cortex. Proc Natl Acad Sci U S A. 2004;101(1):366–371. doi: 10.1073/pnas.2036460100 14695891 PMC314191

[116] Ringach DL, Shapley RM, Hawken MJ. Orientation Selectivity in Macaque V1: Diversity and Laminar Dependence. J Neurosci. 2002;22(13):5639–5651. doi: 10.1523/JNEUROSCI.22-13-05639.2002 12097515 PMC6758222

[117] Vinje WE, Gallant JL. Natural Stimulation of the Nonclassical Receptive Field Increases Information Transmission Efficiency in V1. J Neurosci. 2002;22(7):2904–2915. doi: 10.1523/JNEUROSCI.22-07-02904.2002 11923455 PMC6758304

[118] Hoyer PO. Non-negative Matrix Factorization with Sparseness Constraints. J Mach Learn Res. 2004;5:1457–1469.

[119] Zylberberg J, DeWeese MR. Sparse Coding Models Can Exhibit Decreasing Sparseness while Learning Sparse Codes for Natural Images. PLoS Comput Biol. 2013;9(8). doi: 10.1371/journal.pcbi.1003182 24009489 PMC3757070

[120] Dayan P, Abbott L. Theoretical Neuroscience. MIT Press; 2001.

[121] Sengupta B, Laughlin SB, Niven JE. Balanced Excitatory and Inhibitory Synaptic Currents Promote Efficient Coding and Metabolic Efficiency. PLoS Comput Biol. 2013;9(10). doi: 10.1371/journal.pcbi.1003263 24098105 PMC3789774

[122] Shadlen MN, Newsome WT. The Variable Discharge of Cortical Neurons: Implications for Connectivity, Computation, and Information Coding. J Neurosci. 1998;18(10):3870–3896. doi: 10.1523/JNEUROSCI.18-10-03870.1998 9570816 PMC6793166

[123] Gershon ED, Wiener MC, Latham PE, Richmond BJ. Coding Strategies in Monkey V1 and Inferior Temporal Cortices. J Neurophysiol. 1998;79(3):1135–1144. doi: 10.1152/jn.1998.79.3.1135 9497396

